# Genetically encoded barcodes for correlative volume electron microscopy

**DOI:** 10.1038/s41587-023-01713-y

**Published:** 2023-04-17

**Authors:** Felix Sigmund, Oleksandr Berezin, Sofia Beliakova, Bernhard Magerl, Martin Drawitsch, Alberto Piovesan, Filipa Gonçalves, Silviu-Vasile Bodea, Stefanie Winkler, Zoe Bousraou, Martin Grosshauser, Eleni Samara, Jesús Pujol-Martí, Sebastian Schädler, Chun So, Stephan Irsen, Axel Walch, Florian Kofler, Marie Piraud, Joergen Kornfeld, Kevin Briggman, Gil Gregor Westmeyer

**Affiliations:** 1 Munich Institute of Biomedical Engineering, Department of Bioscience, TUM School of Natural Sciences and TUM School of Medicine, Technical University of Munich, Munich, Germany; 2 Institute for Synthetic Biomedicine, Helmholtz Munich, Neuherberg, Germany; 3 Research Group, Circuits of Birdsong, Max Planck Institute for Biological Intelligence, Martinsried, Germany; 4 Department Circuits–Computation–Models, Max Planck Institute for Biological Intelligence, Martinsried, Germany; 5 Carl Zeiss Microscopy GmbH, ZEISS Group, Oberkochen, Germany; 6 Department of Meiosis, Max Planck Institute for Multidisciplinary Sciences, Goettingen, Germany; 7 Max Planck Institute for Neurobiology of Behavior—caesar (MPINB), Bonn, Germany; 8 Research Unit Analytical Pathology, Helmholtz Zentrum München, Neuherberg, Germany; 9 Helmholtz AI, Helmholtz Munich, Neuherberg, Germany

**Keywords:** Synthetic biology, Nanostructures, Protein design, Cellular neuroscience

## Abstract

While genetically encoded reporters are common for fluorescence microscopy, equivalent multiplexable gene reporters for electron microscopy (EM) are still scarce. Here, by installing a variable number of fixation-stable metal-interacting moieties in the lumen of encapsulin nanocompartments of different sizes, we developed a suite of spherically symmetric and concentric barcodes (EMcapsulins) that are readable by standard EM techniques. Six classes of EMcapsulins could be automatically segmented and differentiated. The coding capacity was further increased by arranging several EMcapsulins into distinct patterns via a set of rigid spacers of variable length. Fluorescent EMcapsulins were expressed to monitor subcellular structures in light and EM. Neuronal expression in *Drosophila* and mouse brains enabled the automatic identification of genetically defined cells in EM. EMcapsulins are compatible with transmission EM, scanning EM and focused ion beam scanning EM. The expandable palette of genetically controlled EM-readable barcodes can augment anatomical EM images with multiplexed gene expression maps.

## Abstract (web summary)

Multiplexable barcodes for electron microscopy are applied to brain imaging.

## Main

Multicolor fluorescent gene reporters have become indispensable biomedical research tools because they provide direct insight into gene expression patterns and can be programmed to report complex cellular states.

Although super-resolution or expansion microscopy approaches can obtain subdiffraction resolution down to nanometer(s), electron microscopy (EM) is still the most established method to routinely achieve (sub)nanometer resolution over imaging volumes with millimeter edge lengths to disentangle subcellular ultrastructural details and cell-to-cell contacts^1–3^.

However, gene reporters for EM remain rare, although they could become as valuable as fluorescent proteins to directly provide multiplexed information on genetically defined cell states with the best available resolution.

Imaging contrast in EM is obtained via well-established fixation and staining protocols involving heavy metal reagents such as osmium tetroxide (OsO_4_), uranyl acetate (UA) and lead citrate (Pb citrate), which provide dense labeling of cellular membranes, organelles, and protein or nucleic acid complexes^4^.

As for genetically controlled EM contrast, the most common method relies on locally triggered polymerization of 3,3′-diaminobenzidine (DAB) to an osmiophilic precipitate, with which OsO_4_ subsequently reacts^5^. DAB polymerization can be initialized by the photochemical generation of radicals catalyzed by the protein miniSOG, as pioneered by Roger Tsien’s group^6^, or by enzymes such as HRP or the engineered APEX/APEX2 enzymes, which also enable proximity labeling^7,8^. However, the enzymatic radical formation and polymerization necessitate specialized protocols to let DAB and the oxidant H_2_O_2_ diffuse into the tissue block.

The polymerization reaction may lead to spatially variable contrast, which can, however, be confined to certain cellular compartments to obtain multiplexed information^9^.

There have also been attempts to accumulate metals directly on genetically encoded proteins bearing tetracysteins or metallothioneins, but these protocols necessitate incubating live cells with toxic metals or laborious post-fixation workflows^10–15^. Moreover, avidin-coated ~15 nm quantum dots have been used as contrast agents that could be targeted to proteins of interest fused to a coiled-coil peptide (VIPER) serving as a bioconjugation tag for a complementary biotinylated coiled-coil^16^. In this mode, receptor-mediated uptake of transferrin receptors or intracellular targets could be visualized by fluorescence and transmission electron microscopy (TEM) as an alternative to immunogold labeling^16^.

We have previously shown that encapsulins can be nontoxically expressed in mammalian cells as self-assembling nanocompartments, which can biomineralize iron-oxide cores of up to 30 nm (refs. ^17,18^). Consequently, they were much more readily visible by TEM and cryo-electron tomography^17,18^ than the 8 nm cores of ferritin^17,18^, expressed as an EM tag named FerriTag^19^.

However, the EM contrast we previously obtained with the native encapsulins in *Drosophila* neurons was minimal, probably due to insufficient availability of ferrous iron in vivo, indicating that the system was not suitable for high-throughput volume EM^18^.

To obtain genetically controlled EM contrast of sufficiently large (tens of nanometers) and geometrically distinct protein assemblies that are easy to use with standard volume EM pipelines, we thus combined heavy metal binders, fluorescent proteins, encapsulins of different icosahedral symmetries, and rigid proteinaceous spacers into EM-readable concentric barcodes, which can augment anatomical EM maps with multiplexed molecular information.

## Results

### Fixation-stable, nanoscale EM contrast

Our first objective was to generate variants of encapsulins, which produce robust EM contrast with nanoscale precision (EMcapsulins) using standard EM fixation and staining protocols, and without additional incubation steps necessary to allow diffusion of DAB and H_2_O_2_ or other exogenous substrates.

Since murine Metallothionein-3 (M) has been demonstrated to be a potent lead binder^20,21^ and acidic stretches of other metallothionein domains have been shown to interact with uranyl ions^22^, we reasoned that M might not only work in specialized EM procedures^10–12,15,23^ but also provide localized EM contrast organized by encapsulins and standard staining protocols.

**Fig. 1:**
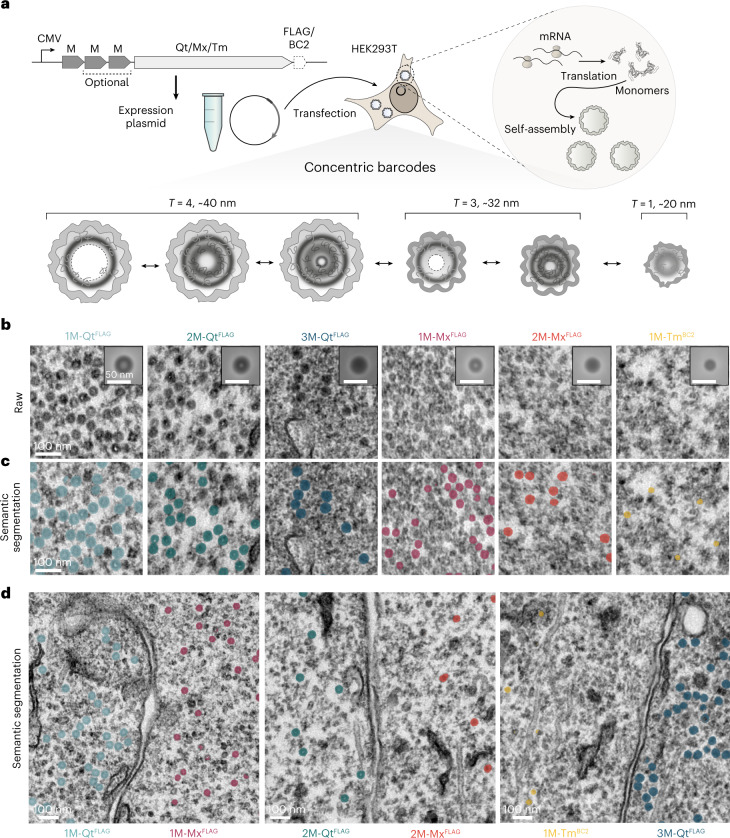
EMcapsulins as fixation-resistant and heavy metal-interacting concentric EM barcodes. **a**, Schematic of the mammalian expression of modular constructs coding for self-assembling encapsulin monomers with N-terminal fusions of metallothioneins (M) and C-terminal surface modifications for targeting (EMcapsulins). Expression of the EMcapsulin variants results in the auto-assembly of hollow protein nanospheres with different triangulation numbers (T), distinct diameters and one to three concatenated copies of M facing the lumen of the porous protein shells. **b**, TEM micrographs (8,000× magnification, 0.5525 pixels nm^−1^) after standard fixation and heavy metal staining EM protocol applied to HEK293T cells expressing the different EMcapsulin variants shown in **a**. Scale bars, 100 nm. Insets show averages of manually segmented image patches (*n* = 1,000 except *n* = 900 for 1M-Tm^BC2^) for each condition (side length of insets: 89.2 nm (49 pixels); scale bar, 50 nm). **c**, Multiclass semantic segmentation from the end-to-end U-net architecture. **d**, Multiplexed detection of different EMcapsulin class combinations in adjacent HEK293T cells with overlaid semantic segmentation masks. Scale bars, 100 nm.

Given M’s small size and flexibility, we thought it unlikely to disrupt encapsulin assembly. We thus generated direct fusions to the lumen-facing N-terminus of encapsulin monomers instead of encapsulating M as cargo protein^17,18^ to obtain better control over the stoichiometry and suppress background from nonencapsulated cargo (Fig. 1a).

**Extended Data Fig. 1:**
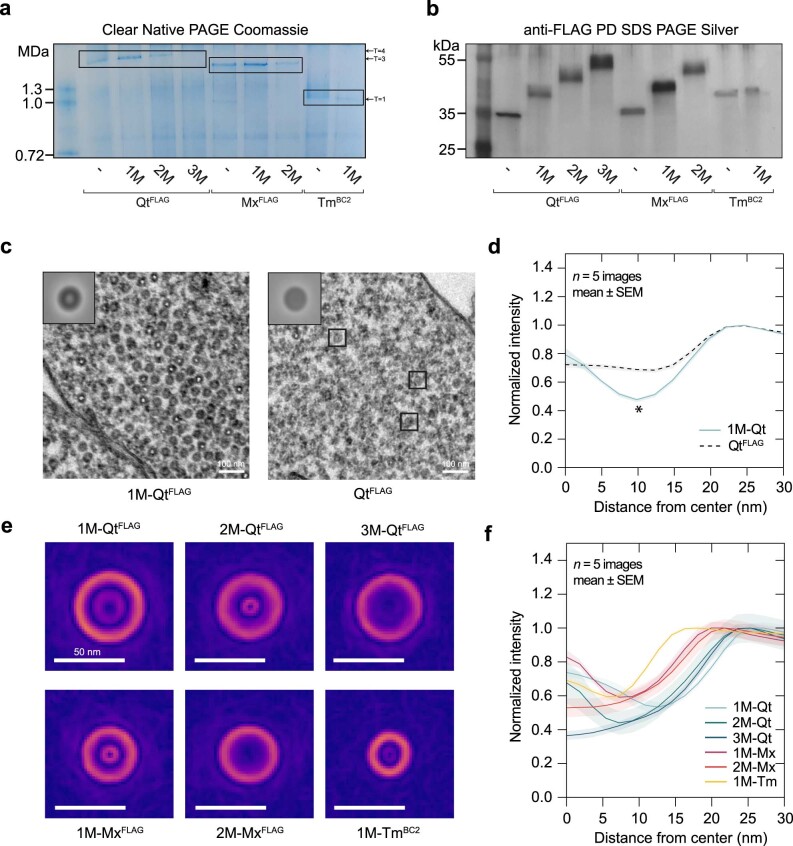
Biochemical characterization and TEM analysis of the EMcapsulin classes. **a**, Clear-Native (CN) PAGE analysis of lysates from HEK293T cells expressing six EMcapsulin variants (1M-Qt^FLAG^, 2M-Qt^FLAG^, 3M-Qt^FLAG^, 1M-Mx^FLAG^, 2M-Mx^FLAG^, 1M-Tm^BC2^) in comparison to wild-type encapsulin shells (Qt^FLAG^, Mx^FLAG^, (CC)-Tm^BC2^) giving distinct high molecular weight bands corresponding to *T*=4, *T*=3, and *T*=1 icosahedral symmetries. Please note that the T=4 band corresponding to 3M-Qt^FLAG^ has a significantly decreased visibility. **b**, Corresponding silver-stained SDS-PAGE loaded with the same EMcapsulins as in a, pulled down with either anti-FLAG or anti-BC2 beads. The expected size shifts of the fusion proteins (1M-Qt^FLAG^: 40.6 kDa, 2M-Qt^FLAG^: 48.3 kDa, 3M-Qt^FLAG^: 55.0 kDa, 1M-Mx^FLAG^: 40.1 kDa, 2M-Mx^FLAG^: 47.7 kDa, 1M-Tm^BC2^: 39.4 kDa) were detected as compared with the wild-type encapsulins (Qt^FLAG^: 33.4 kDa, Mx^FLAG^: 31.9 kDa). **c**, Comparison of TEM micrographs from HEK293T cells expressing 1M-Qt^FLAG^ and wild-type Qt^FLAG^. The insets show average intensity projections of 1000 particles. The scale bar is 100 nm, and the bounding box is 89.2 nm. **d**, Average radial profile plots (mean±SEM from 5 TEM images, with 50 segmented EMcapsulins per image) corresponding to c, showing the relative signal reduction in the lumen of 1M-Qt^FLAG^ as compared with wild-type Qt^FLAG^ encapsulin. Image intensities were normalized to the values of the surrounding cytosol. * indicates significantly different intensities at 9.85 nm from the center (P < 0.0001, two-tailed t-test). **e**, Sobel-filter applied on the average intensity projections from 5 TEM images, with 50 segmented EMcapsulins for each of the 6 EMcapsulin classes yielding mean diameters of 42.32 ± 1.44 nm for 1M-Qt^FLAG^, 39.08 ± 1.44 nm for 2M-Qt^FLAG^, 40.16 ± 0.72 nm for 3M-Qt^FLAG^, 32.24 ± 1.35 nm for 1M-Mx^FLAG^, 34.4 ± 1.61 nm for 2M-Mx^FLAG^ and 24.22 ± 1.44 nm for 1M-Tm^BC2^ (mean±SEM). To determine diameters from Sobel-filter applied images, central line plots in x and y were averaged, and the distance between maxima corresponding to the edges was obtained for the five replicates. **f**, Average radial plot profiles of the 6 EMcapsulin classes (mean±SEM from 5 TEM images, with 50 segmented EMcapsulins per image). Source data

When we expressed M as N-terminal fusions to encapsulins from *Quasibacillus thermotolerans* (Qt), *Myxococcus xanthus* (Mx) and *Thermotoga maritima* (Tm) in HEK293T, we could confirm by Clear Native (CN) polyacrylamide gel electrophoresis (PAGE) that Qt^FLAG^ and Mx^FLAG^ variants showed the expected electrophoretic running behavior for icosahedral assemblies with the triangulation numbers (T) *T* = 4 and *T* = 3 under native conditions, irrespective of the N-terminal fusion of M (Extended Data Fig. 1a).

1M-Tm^BC2^ showed similar electrophoretic mobility as the *T* = 1 assembly of Mx^FLAG^, which is known to occur when no cargo proteins are co-expressed^17,24^. Corresponding silver-stained SDS polyacrylamide gel analysis of the fusion proteins pulled down from cell lysates showed weight shifts consistent with the calculated weights compared to the unmodified encapsulin monomers (Extended Data Fig. 1b).

On the basis of these promising biochemical data, we next expressed the 1M-Qt^FLAG^ fusion and wild-type encapsulin (Qt^FLAG^) in HEK293T cells and subjected them to a standard EM sample preparation protocol, consisting of fixation with glutaraldehyde and post-fixation with OsO_4_, followed by heavy metal staining with UA and lead citrate, epon embedding, microtome sectioning and imaging on TEM grids.

The resulting TEM micrographs showed annular contrast shapes with a brighter center spot outlined by a darker ring for 1M-Qt^FLAG^.

In distinction, the wild-type encapsulins without M (Qt^FLAG^) were only minimally contrasted against the cytosolic background, as can also be appreciated from the average radial profiles (Extended Data Fig. 1c,d).

### Concentric EM barcodes

We next sought to evaluate whether concatenating multiple copies of M on the inner surface of EMcapsulins could add distinct layers of annular EM contrast **(**Fig. 1a). Indeed, in comparison with the TEM contrast of 1M-Qt^FLAG^, we observed a broadening of the contrasted ring for 2M-Qt^FLAG^ and the vanishing of the bright spot in the center when a third metallothionein domain (3M-Qt^FLAG^) was added (Fig. 1a,b). Since concatenation of three murine metallothionein sequences prevented EMcapsulin assembly, we constructed 3 M from a chimeric sequence of three metallothionein domains from different species (MmMT3 (ref. ^20^), SeSmtA^22^ and TaEC1 (ref. ^25^)).

In the case of Mx^FLAG^, the outer diameter of the contrasted edges was expectedly smaller than that of Qt^FLAG^ such that a single M (1M-Mx^FLAG^) resulted in a bright center similar in diameter to that seen for 2M-Qt^FLAG^. Adding a second M to Mx^FLAG^ (2M-Mx^FLAG^) abolished the bright central spot. Complementarily, 1M-Tm^BC2^ exhibited the smallest outer diameter without a prominent bright center (Fig. 1b).

Thus, the modular combination of metal interactors and different-sized protein shells led to well-separated outer diameters for the three types of spherical protein shells and distinct radial profiles for all six classes of EMcapsulins (Extended Data Fig. 1e,f).

Higher TEM magnifications demonstrate that the layering of contrasted rings is in line with the location of M on the inner surface of the protein shell (Supplementary Fig. 1).

### Automatic semantic segmentation

To test how robustly the EMcapsulins can be identified and classified, we trained an end-to-end U-net^26^ for multiclass semantic segmentation of the six EMcapsulin classes on 250 TEM images obtained from all experimental categories reported in this manuscript. Semantic segmentation results are shown as overlays in Fig. 1c, color-coded by class as defined in Fig. 1a. Segmentation metrics, such as the Dice similarity coefficients (DSC) per EMcapsulin class, are tabulated in Supplementary Table 1, giving an average DSC score of 0.65 (validation set), 0.71 (test set 1) and 0.63 (test set 2).

We also report several object-detection metrics, such as an average recognition quality (harmonic mean of precision and recall^27^) of 0.64, 0.70 and 0.61 for the validation set and test sets 1 and 2, respectively.

For comparison, we also implemented a sequential segmentation (U-net^26^) followed by classification (EfficientNetV2-M^28^) of the segmented image patches after background subtraction to ensure that the classification signal emerges from the EMcapsulins themselves (Supplementary Fig. 2a). Segmentation and classification scores compared with human annotations are given in Supplementary Fig. 2b–e.

**Extended Data Fig. 2:**
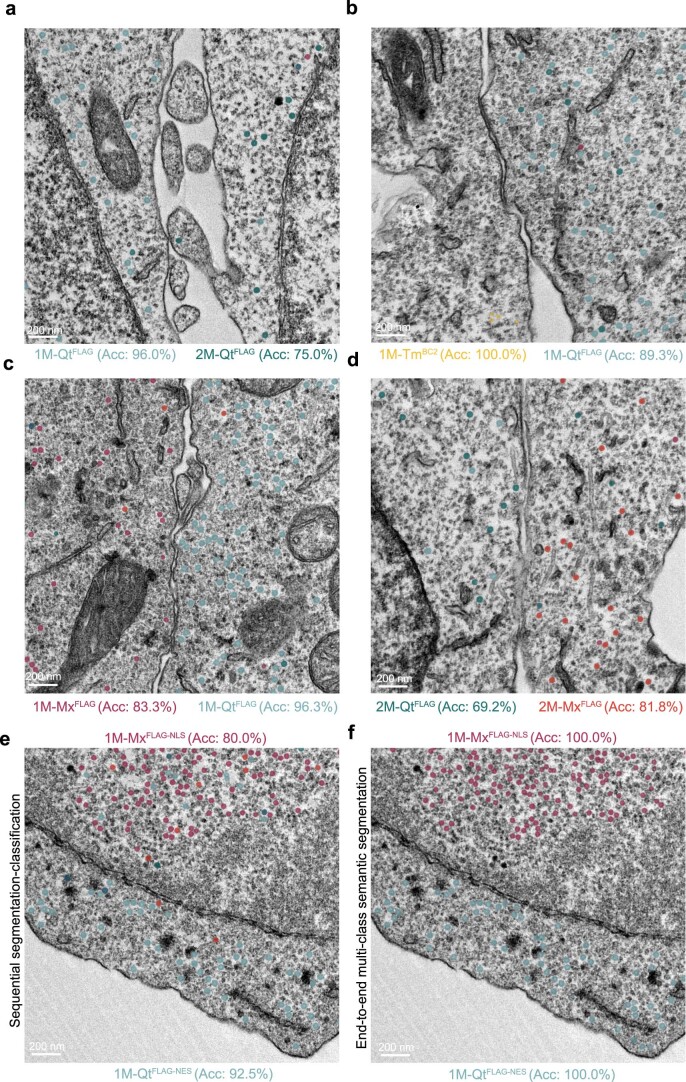
Results for multiplexed EMcapsulin detection by the sequential segmentation-classification pipeline. **a-d** Examples of multiplexed detection of two different EMcapsulins classes in adjacent HEK293T cells color-coded as defined in Fig. 1. The scale bars represent 200 nm. **e,f** Multiplexed detection of 1M-Qt^FLAG-NES^ in the cytosol and 1M-Mx^FLAG-NLS^ in the nucleus within the same HEK293T cell, with overlays in e, generated by the sequential segmentation-classification pipeline and overlays in f, generated using the end-to-end multi-class semantic segmentation network as shown in Fig. 1. The scale bar represents 200 nm.

When we then expressed combinations of two EMcapsulin classes in separate HEK cells, pooled in the same sample for multiplexed detection, we found that the end-to-end network (Fig. 1d and Extended Data Fig. 2a–d) produced slightly better semantic segmentation results than the sequential segmentation-classification pipeline (Extended Data Fig. 2e,f).

For optimal usability, we also generated a napari^29^ graphical user interface (GUI) that allows for interactive curation of semantic segmentations using the publicly available pretrained models (Supplementary Fig. 3).

### Programmable EMcapsulin nanopatterns

We next aimed to further increase the encoding capacity of the multiplexed EM gene reporters by arranging multiple EMcapsulins into different patterns.

**Fig. 2:**
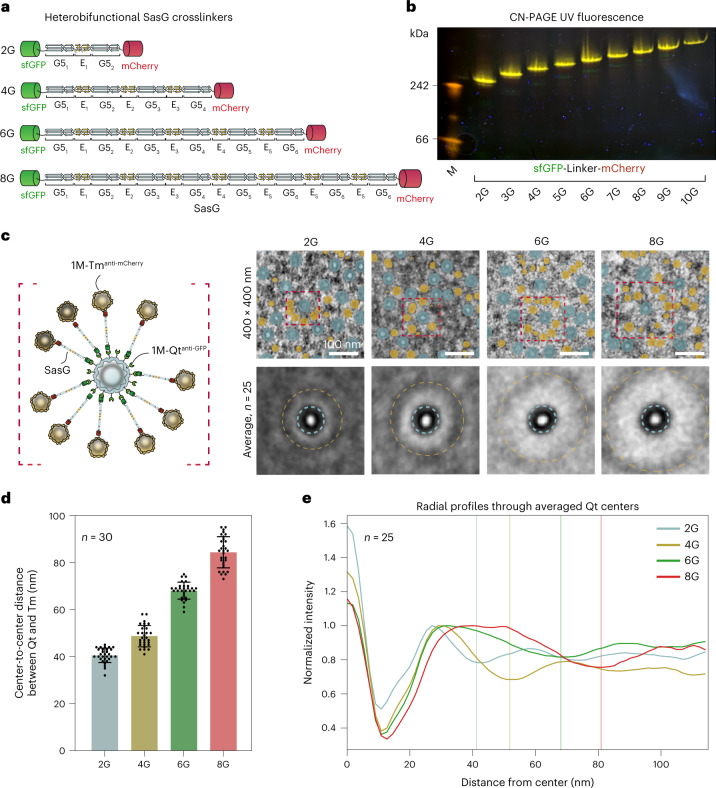
Modular EMcapsulin patterns. **a**, A rigid heterobifunctional cross-linker was constructed from SasG capped off with sfGFP and mCherry. Different linker lengths were obtained by increasing the number of G5 domains connected by E domains (sfGFP-2G-mCherry up to sfGFP-10G-mCherry). **b**, CN PAGE under UV illumination loaded with lysates from HEK293T cells expressing sfGFP-SasG-mCherry heterobifunctional linkers (2–10G units) yielded discrete yellow fluorescent bands. **c**, Co-expression of 1M-Qt^anti-GFP^ and 1M-Tm^anti-mCherry^ with indicated SasG cross-linkers (2–8G units) resulted in distinct EMcapsulin patterns with ~40 nm ring-shaped centers from 1M-Qt^anti-GFP^ surrounded by ~25 nm spherical objects (1M-Tm^anti-mCherry^) in TEM micrographs. The upper panel shows 400 × 400 nm exemplary regions showing the concentric, programmable EMcapsulin patterns with overlaid semantic segmentation results from the end-to-end network (8,000× magnification, 0.5525 pixels nm^−1^). Scale bars, 100 nm. The lower panel shows averages around selected Qt centers surrounded by a layer of Tm (*n* = 25). The bounding box has a side length 165 nm. **d**, Distances between the centers of 1M-Qt^anti-GFP^ and the centers of surrounding 1M-Tm^anti-mCherry^ for the indicated cross-linker lengths (*n* = 30); error bars, ±s.d. **e**, Average radial profile plots from the center of each 1M-Qt^anti-GFP^ (*n* = 25) outwards via the cross-linkers towards the surrounding ring of 1M-Tm^anti-mCherry^ color-coded for cross-linker length. The vertical lines represent contrast minima generated by the surrounding 1M-Tm^anti-mCherry^. Source data

We thus generated a series of rigid heterobifunctional crosslinkers from the microbial filamentous protein SasG^30,31^ capped off with the fluorescent proteins superfolder GFP (sfGFP) and mCherry, for which bioorthogonal intrabodies^32,33^ exist (Fig. 2a).

**Extended Data Fig. 3:**
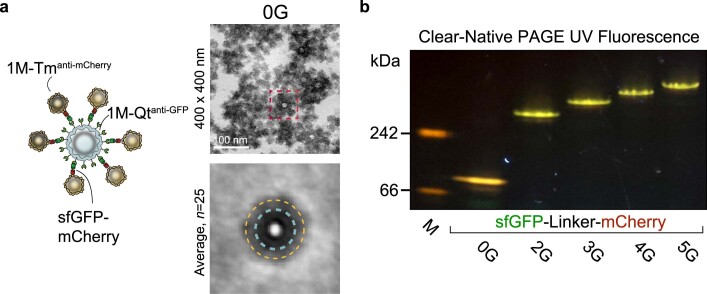
Additional linker lengths for generating EMcapsulin patterns. **a**, TEM micrograph, and the average of the 0 G control condition (direct fusion of sfGFP and mCherry), which is also depicted in the schematic. The scale bar represents 100 nm. The bounding box showing the average projection represents 165 nm. The distance from Qt to Tm center is 32.83 ± 2.06 nm (mean±SD). **b**, Clear Native PAGE under UV illumination loaded with lysates of HEK293T expressing a direct fusion of sfGFP and mCherry (0 G) as a control, as well as 2G-5G SasG linkers. The redshift observed for the 0 G band is due to FRET between the closely linked sfGFP and mCherry. Source data

We obtained increasing linker lengths by concatenating G5 domains connected via E domains^30,31^ (2–8G) that resulted in distinct protein species as shown by the sharp yellow fluorescent bands on CN PAGE upon UV illumination (Fig. 2b). Only a direct fusion of the fluorescent proteins without SasG (sGFP-0G-mCherry) emitted at a red-shifted wavelength, indicating Förster resonance energy transfer (Extended Data Fig. 3).

When we then co-expressed the fluorescent linkers together with different EMcapsulins that were surface-modified with the matching intrabodies (1M-Qt^anti-GFP^ and 1M-Tm^anti-mCherry^), we observed distinct EMcapsulin patterns in TEM, in which the annular 1M-Qt shapes were surrounded by 1M-Tm at distinct interparticle distances dependent on the linker length (Fig. 2c–e).

### Dual EM and fluorescent gene reporters

**Fig. 3:**
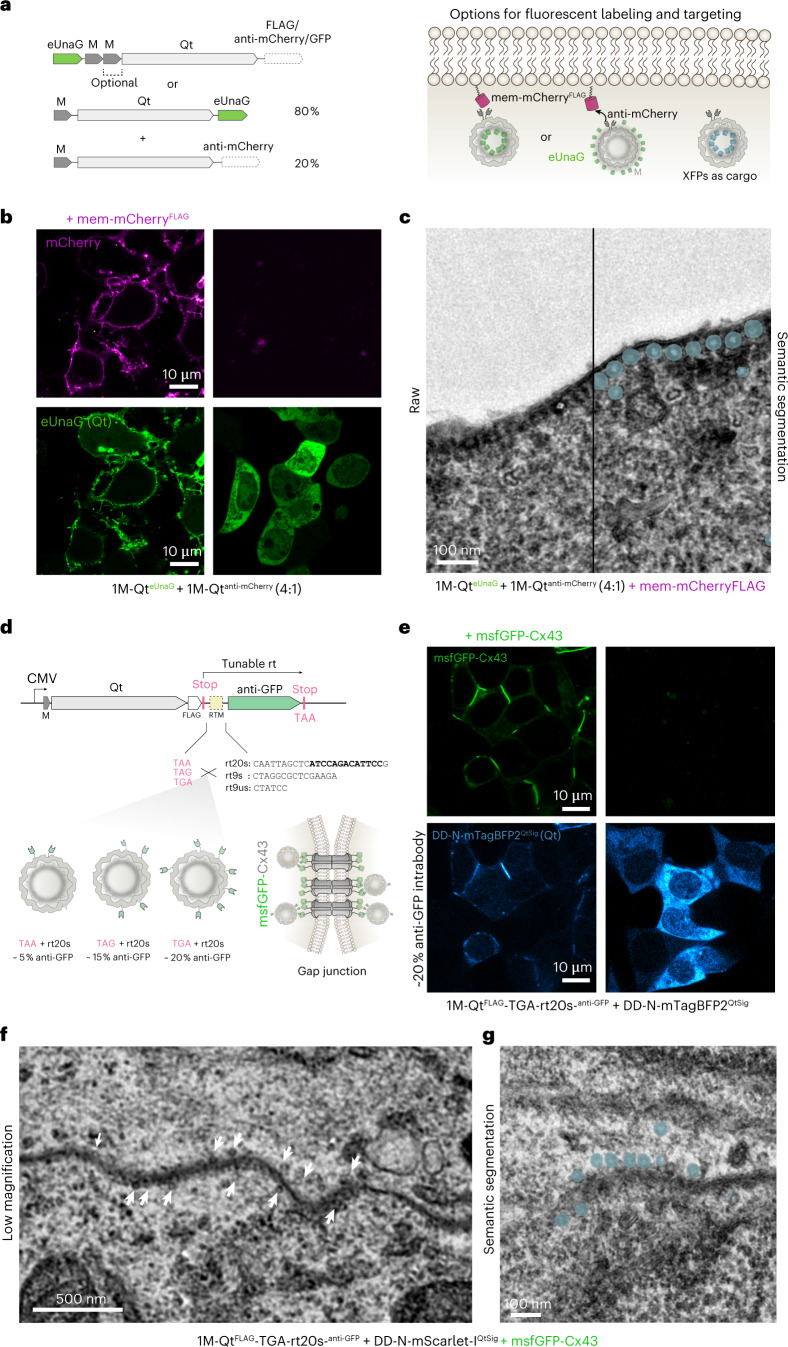
Targetable fluorescent EMcapsulins as high-contrast labels for CLEM. **a**, Targetable fluorescent EMcapsulins can be generated by N-terminally appending the small monomeric fluorescent protein (eUnaG) to 1M- and 2M-Qt, which harbor surface-exposed anti-mCherry intrabodies. Alternatively, EMcapsulin monomers fused to outward-facing eUnaG can be co-expressed with EMcapsulin monomers, a fraction of which can harbor an intrabody. **b**, Confocal laser scanning microscopy of fluorescent EMcapsulin composed of 1M-Qt^eUnaG^ monomers co-expressed at a ratio of 4:1 with monomers harboring anti-mCherry intrabodies (1M-Qt^anti-mCherry^) with and without co-expression of membrane-bound mCherry (mem-mCherry^FLAG^). Scale bar, 10 µm. **c**, Corresponding TEM micrograph of mem-mCherry-targeted 1M-Qt^anti-mCherry^ + 1M-Qt^anti-mCherry^ (4:1). Scale bars, 100 nm. **d**, Control over the ratios of different EMcapsulin monomers can be achieved via tunable ribosomal rt cassettes encoded on a single cistron. To this end, different combinations of stop codons and short rt-promoting motifs are combined at the end of the ORF encoding 1M-Qt. In case rt occurs, the C-terminus of 1M-Qt^FLAG^ is further extended by an anti-GFP intrabody. **e**, Confocal fluorescence microscopy of HEK293T co-expressing the gap junction forming protein msfGFP-Cx43 together with the 1M-Qt^FLAG^-TGA-rt20s-^anti-GFP^ yielding ~20% anti-GFP intrabodies on the EMcapsulin surface. The EMcapsulins variants are also rendered fluorescent via co-expression of mTagBFP2 as cargo proteins harboring the Qt encapsulation signal (QtSig) and an N-terminal degron (DD-N), leading to the degradation of nonencapsulated fluorescent proteins. Scale bars, 10 µm. **f**,**g**, Corresponding TEM micrographs showing 1M-Qt (with ~20 % anti-GFP intrabody) particles labeling gap junctions (**f**), a low-magnification view (scale bar, 500 nm; white arrowheads point to 1M-Qt^FLAG^ EMcapsulins) and a higher-magnification view (scale bar, 100 nm) (**g**). Multiclass semantic segmentation results are shown color-coded in **g**, as defined in Fig. 1.

While direct fusions of fluorescent proteins of the GFP family tended to disrupt encapsulin assembly in our hands, we reasoned that the flexible M might function as a linker to a small fluorescent protein such as eUnaG to allow for proper assembly^34^. The resulting dual-contrast EMcapsulins could be targeted to subcellular structures of interest for sequential analysis by fluorescent and electron microscopy (Fig. 3a).

**Extended Data Fig. 4:**
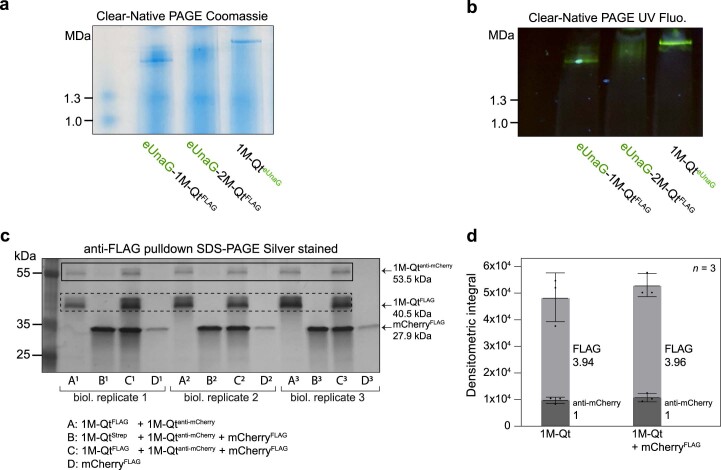
Characterization of targeted dual-mode fluorescent EMcapsulin variants. **a**, Coomassie-stained Clear-Native PAGE loaded with lysates of HEK293T cells expressing dual-mode fluorescent EMcapsulin variants eUnaG-1M-Qt^FLAG^, eUnaG-2M-Qt^FLAG^ or 1M-Qt^eUnaG^. **b**, Unstained UV-illuminated CN-PAGE shown in a. **c**, Silver-stained SDS PAGE after anti-FLAG pull-down from lysates of HEK293T co-expressing the combinations A, B, and C as specified in the figure. The relative DNA amounts of 1M-Qt^FLAG^ or Strep to 1M-Qt^anti-mCherry^ were kept constant at 4:1. **d**, Densitometric analysis of the conditions A and C shown in c, giving the relative amounts of 1M-Qt^FLAG^ with respect to 1M-Qt^anti-mCherry^ (set to 1) obtained from 3 biological replicates (with three technical replicates, mean±SD). Source data

Indeed, eUnaG-1M-Qt^FLAG^ migrated as a well-defined band on CN PAGE corresponding to an assembly with *T* = 4 icosahedral symmetry (Extended Data Fig. 4a,b). In comparison, a blurred band was detected for eUnaG-2M-Qt^FLAG^, indicating more heterogeneous assemblies, possibly due to space limitations in the lumen of the nanospheres (Extended Data Fig. 4a,b).

We thus investigated whether eUnaG is also tolerated on the outer surface of Qt, thus reserving the EMcapsulin lumen for variable copies of M (Fig. 3a). This variant indeed showed a sharp fluorescent band with decreased electrophoretic mobility as compared with eUnaG-1M-Qt^FLAG^ in agreement with the expected increase in the hydrodynamic diameter from adding proteins on the outer surface (Extended Data Fig. 4a,b).

### Labeling of subcellular targets

Next, we wanted to test how well the dual-modality EMcapsulins could be directed to intracellular locations of interest. We, therefore, installed anti-mCherry-intrabodies on the outer surface of the fluorescent EMcapsulin (1M-Qt^eUnaG^) by co-expressing 1M-Qt^anti-mCherry^ in a ~4:1 ratio (Extended Data Fig. 4c,d).

When we co-expressed membrane-targeted mCherry (mem-mCherry^FLAG^), the fluorescent EMcapsulins co-localized to the membrane (Fig. 3b) (Manders’ coefficient M1 0.870, Manders’ M2 0.966, Costes *P* value 1.00), and the corresponding TEM images could resolve individual EMcapsulins lined up on the membrane (Fig. 3c).

**Extended Data Fig. 5:**
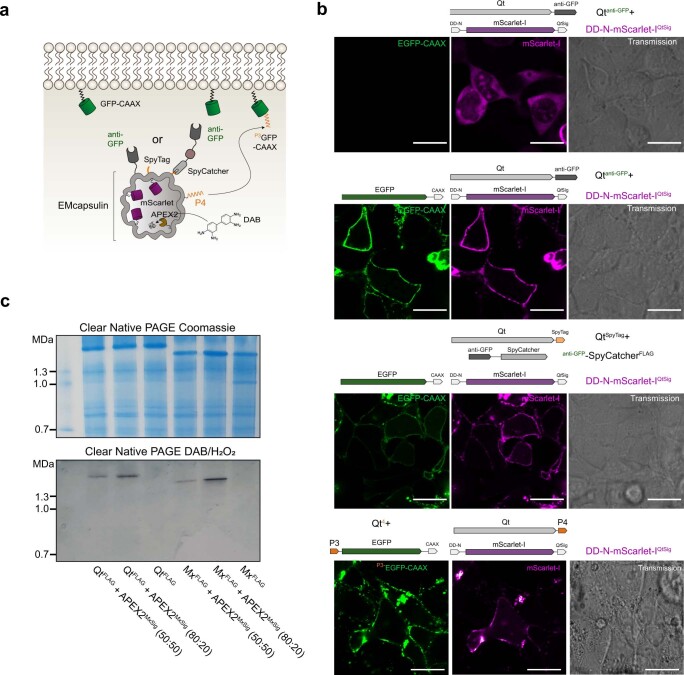
Alternative variants of fluorescent, targetable EMcapsulins. **a**, Genetic constructs and schematics of targetable EMcapsulins as alternative options to those shown in Fig. 3. Instead of a direct C-terminal fusion, intrabodies can also be covalently attached via SpyTag/SpyCatcher chemistry. Alternatively, EMcapsulins can be addressed to target proteins with bio-orthogonal coiled-coil (CC) pairs, enabling intracellular targeting. Instead of the direct N-terminal fusion of eUnaG, fluorescence is obtained here by mScarlet-I, which is degraded via a degron (DD) unless it is encapsulated to the encapsulin lumen via an encapsulation signal (QtSig). **b**, Exemplary confocal fluorescence microscopy images with the membrane target shown in green (EGFP-CAAX), except for the control in the first row and the respective targetable EMcapsulin variants loaded with mScarlet-I in magenta. Scale bars represent 20 µm. **c**, APEX2 co-expressed as cargo in Qt and Mx for optional DAB-polymerization as shown on Clear Native PAGE. The upper panel shows the Coomassie-stained gel with bands corresponding to the assembled nanocompartments, whereas the lower panel shows the same samples applied to a second gel incubated with DAB and hydrogen peroxide resulting in brown/black bands for the nanocompartments with polymerized DAB. Source data

To showcase the modularity of the labeling approaches, we tested the expression of SpyTag/SpyCatcher^35^ adapters or bioorthogonal coiled-coil pairs^36^ as targeting moieties and fluorescent proteins or APEX2 (ref. ^8^) as cargo proteins (Extended Data Fig. 5).

On the basis of these promising results, we next chose connexins as a molecular target for fluorescent EMcapsulins. Connexins assemble into connexons forming gap-junctions that contribute to cell-to-cell communication in many biological systems, including in neuronal networks^37,38^.

Connexins have also previously been fused to fluorescent proteins^8,39,40^ and modified with tetracysteine tags targeted by biarsenical fluorophores, including ReAsH. These were then used for photo-induced production of singlet oxygen to polymerize DAB, leading to electron-dense precipitates on the gap junctions, validated by immunogold labeling^41^.

**Extended Data Fig. 6:**
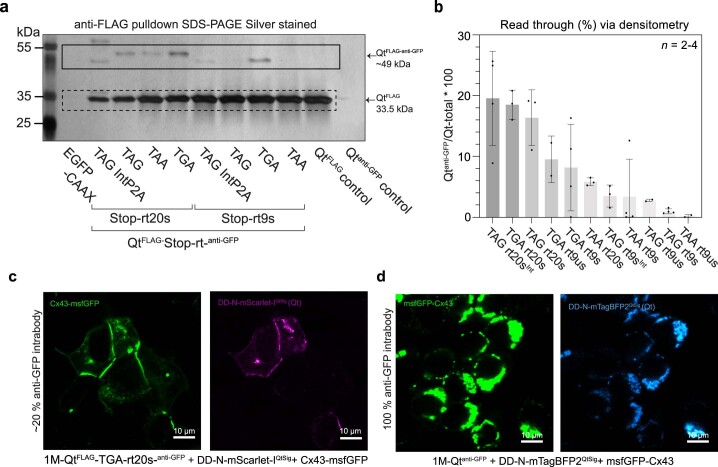
Quantification of tunable translational read-through and Connexin 43 targeting. **a**, Exemplary densitometric quantification of the fractional read-through via different combinations of stop codons and read-through motifs (related to main Fig. 3). Read-through resulted in the extension of the FLAG tag with an anti-GFP intrabody (higher molecular weight band). The heterotypic EMcapsulins were pulled down via the FLAG tag. Note that the exemplary SDS-PAGE shown here does not contain stop codon combinations with RT9us. The more complex band pattern in the case of TAG IntP2A indicates intein splice patterns. The lower band can be explained by correct splicing resulting in a 46.9 kDa band (Qt^anti-GFP^). The higher band can be explained by incorrect splicing, resulting in Qt^anti-GFP^ fused to IntP2A with a size of 68.5 kDa. **b**, Percent read-through (rt) determined from densitometric analysis of the respective SDS-PAGE bands (Qt^FLAG-Linker-anti-GFP^ / Qt-total * 100), (The bars represent the mean±SD). **c**, Alternative labeling of Cx43 with C-terminal fusion of Cx43-msGFP (as opposed to N-terminal fusion as in Fig. 3e) and corresponding confocal fluorescence microscopy images. The EMcapsulins were made fluorescent via co-expression of mScarlet-I as cargo proteins. Scale bars represent 10 µm. **d**, Control condition for the experiment shown in Fig. 3e in which 100% of anti-GFP intrabody, that is, 240 copies were expressed per EMcapsulin, leading to an agglomeration of the msfGFP-Cx43 upon EMcapsulin binding. Scale bar is 10 µm. Source data

To gain control over the stoichiometry of our fully genetic system from a single genetic construct, we developed a translational read-through (rt) system based on shortened variants of previously identified rt motifs^42^ (Fig. 3d), in which the nascent amino-acid chain of an EMcapsulin is released at a leaky stop codon in the majority of cases. In contrast, the ribosome continues translating over the adjacent rt motif in a tunable fraction of cases to also translate the fused C-terminal intrabody. By combining three stop codons with three shortened rt motifs (rt20s, rt9s and rt9us), we created a small library yielding rt efficiencies between ~1% and ~20% (Extended Data Fig. 6a,b).

When co-expressing the rt cassette yielding ~20% translational rt, that is, ~50 intrabodies per EMcapsulin (1M-Qt^FLAG^-TGA-rt20s-^anti-GFP^) with msfGFP-Cx43, we found proper transport of Cx43 to the membrane (green channel) and adequate labeling by EMcapsulins (blue channel) on the membrane (Fig. 3e) (Manders’ coefficient M1 0.989, Manders’ coefficient M2 0.953, Costes *P* value 1.00), which was confirmed in the TEM micrographs from corresponding samples (Fig. 3f,g). The same rt cassette (TGA-rt20s) yielded similar connexin labeling also for Cx43 with a C-terminally fused msfGFP (Extended Data Fig. 6c). A control condition without an rt cassette, that is, expressing 100% intrabodies per 1M-Qt (240 copies on the surface) resulted in clustering of Cx43 in the ER and no clear cell surface signal (Extended Data Fig. 6d).

To further demonstrate the value of fluorescent EMcapsulins, we performed live-cell microscopy in mammalian oocytes co-expressing different mCherry-tagged targets with eUnaG-1M-Qt^anti-mCherry^ (Supplementary Fig. 4a). Green fluorescent EMcapsulin colocalized with mCherry-RAB11A on recycling endosomes (Supplementary Fig. 4b–d and Supplementary Videos 2 and 3) or with mCherry-Myo5b on cargo vesicles (Supplementary Fig. 4e–g and Supplementary Video 5), in line with previously reported subcellular localizations^43^. Automated segmentation and tracking analyses revealed no substantial difference in the volume and speed of labeled compartments in the absence **(**Supplementary Videos 1 and 4) or presence of EMcapsulins. In contrast, a homogeneous background was observed in the absence of mCherry targets (Supplementary Fig. 4b,e). In addition, we targeted mCherry-PLK1 to label relatively static acentriolar microtubule-organizing centers (Supplementary Fig. 4e).

### Multiplexed EMcapsulin contrast in *Drosophila* neurons

**Fig. 4:**
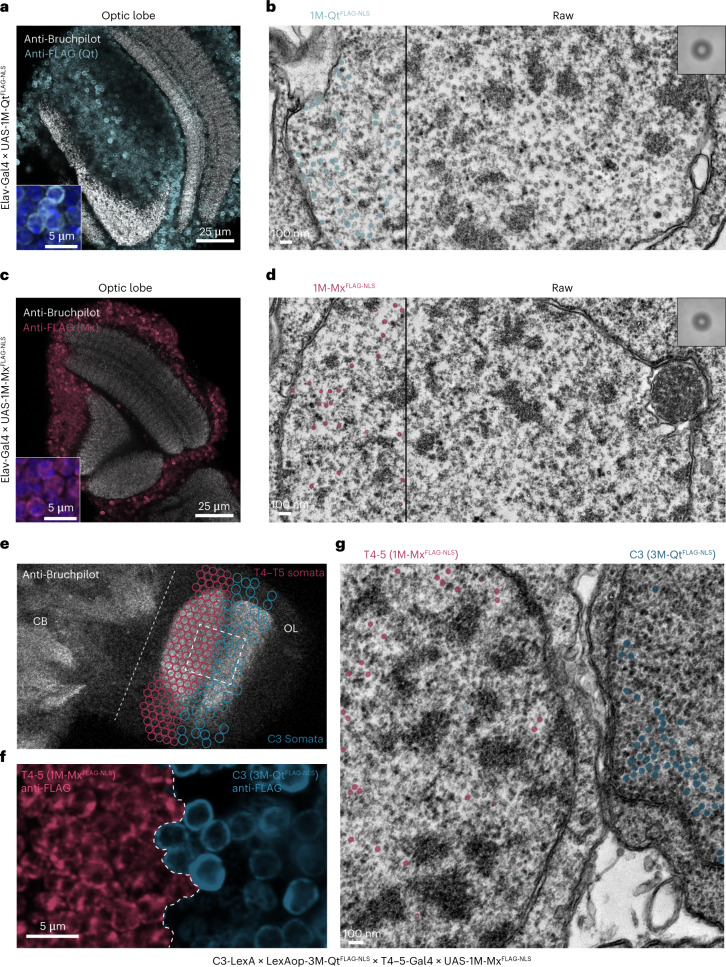
EMcapsulins as multichannel gene reporters in *Drosophila* neurons. **a**,**c**, Overview fluorescence confocal microscopy images of the optic lobe (OL) of a *Drosophila* line with pan-neuronal expression of 1M-Qt^FLAG-NLS^ (**a**) or 1M-Mx^FLAG-NLS^ (**c**) harboring an NLS. FLAG epitopes on the exterior surface of EMcapsulins (anti-FLAG, cyan for 1M-Qt^FLAG-NLS^ and red for 1M-Mx^FLAG-NLS^) are colocalized with nuclei (inset with DAPI in blue; scale bar, 5 µm), but do not exhibit cytoplasmic expression (anti-Bruchpilot, gray). Scale bar, 25 µm. **b**,**d**, Corresponding TEM micrographs with semantic segmentation maps as overlays. Scale bar, 100 nm. The insets show the average of the respective EMcapsulin class identified in the validation dataset (*n* = 132, bounding box: 89.2 × 89.2 nm). **e**, Overview fluorescence confocal microscopy image of the central brain (CB) and optic lobe (OL). **f**, Zoom-in to the *Drosophila* optic lobe containing both T4–T5 and C3 somata, expressing 1M-Mx^FLAG-NLS^ and 3M-Qt^FLAG-NLS^ EMcapsulins. **g**, Corresponding TEM micrograph with overlaid multiclass semantic segmentation masks color-coded as in Fig. 1. Scale bar, 100 nm.

We next sought to assess whether the EMcapsulin contrast in cell culture would also transfer to in vivo applications in neurons. We thus generated transgenic *Drosophila* lines with pan-neuronal expression of 1M-Qt^FLAG-NLS^ and 1M-Mx^FLAG-NLS^ harboring a nuclear localization signal (NLS) (Fig. 4), whose functionality was confirmed beforehand in cell culture (Supplementary Fig. 5) via immunohistochemical analyses (insets in Fig. 4a,c).

Higher TEM magnifications again showed increased contrast on the inner surface of the EMcapsulin protein shells, similar to what was observed in HEK cells (Supplementary Fig. 6).

To demonstrate multiplexed EMcapsulin detection in different neuronal types in the same animal, we generated a transgenic *Drosophila* line expressing 3M-Qt^FLAG-NLS^ in C3 neurons and 1M-Mx^FLAG-NLS^ in T4–5 neurons, whose somata are adjacent in the optic lobe, such that one can capture them in the same field of view in TEM (Fig. 4e–g). Furthermore, we have co-expressed 1M-Qt with a nuclear export signal (1M-Qt^FLAG-NES^) and 1M-Mx^FLAG-NLS^ in the same neuronal type and observed substantial expression of 1M-Qt^FLAG-NES^ in neuronal processes (Supplementary Fig. 7).

### EMcapsulin contrast in volume SEM

**Fig. 5:**
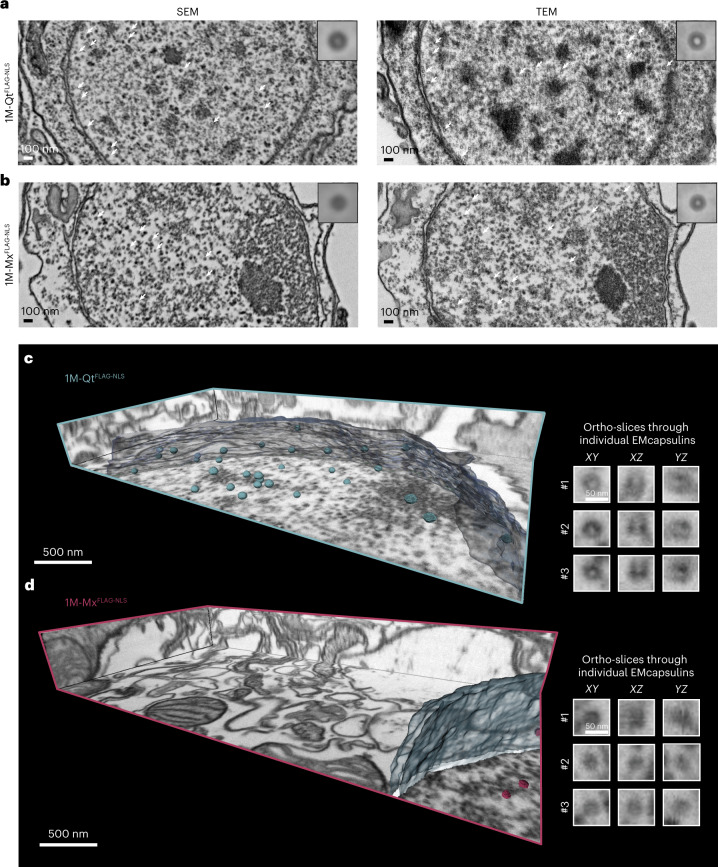
EMcapsulin contrast in SEM and FIB-SEM of *Drosophila* neurons. **a**,**b**, SEM and corresponding TEM micrographs of the identical sample from a *Drosophila* line with pan-neuronal expression of either 1M-Qt^FLAG-NLS^ (**a**) or 1M-Mx^FLAG-NLS^ (**b**) after a standard fixation and staining protocol. Ultrathin sections were captured either on TEM grids or on silica wafers for subsequent analysis by TEM and SEM (inverted contrast), allowing for the analysis of similar cuts through the identical cell with both techniques. SEM images were obtained from a Zeiss GeminiSEM with sense-BSD, Tandem Decel with 1.5 kV. Corresponding TEM images were acquired on a Zeiss Libra120 at 120 kV, 13 µA and 100 µrad. Insets show averages (*n* = 30) of the respective particle from manual segmentation. White arrowheads indicate the presence of EMcapsulin particles inside the nucleus. **c**,**d**, Isotropic FIB-SEM image volumes (4 nm voxel size) of *Drosophila* brains expressing 1M-Qt^FLAG-NLS^ (cyan) (**c**) and 1M-Mx^FLAG-NLS^ (red) (**d**) targeted to the nucleus. EMcapsulins and nuclear membranes were manually segmented and rendered within the FIB-SEM volume bounded by the ortho-slices. The magnifications (right) show ortho-slices through three EMcapsulins. Volume acquisition was performed with an SEM beam voltage of 1.3 kV and a working distance of 5 mm, at a nominal voxel size of 4 nm using an InLens detector. The FIB Ga beam was accelerated by 30 kV voltage at a current of 700 pA. Scale bars, 500 nm (overview) and 50 nm (zoom-ins). Please also see Supplementary Video 6.

Since scanning electron microscopy (SEM) is the other common mode for volume EM, we compared SEM and TEM contrast directly from adjacent ultramicrotome sections of the same *Drosophila* neurons. We found that similar image information can be obtained from EMcapsulins in TEM and SEM (Fig. 5a, b). The line profiles through the EMcapsulins in TEM and SEM show similar outer diameters based on the contrast edges, whereas the lumen of both nanospheres appears brighter in TEM than in SEM (Supplementary Fig. 8).

We also tested whether the EMcapsulin contrast is compatible with focused ion beam (FIB)-SEM tomography^44^, which enables fully automated volume imaging with isotropic nanometer resolution. We chose a voxel size of 4 nm, a resolution informative for connectome analyses and could readily discern 1M-Qt^FLAG-NLS^ (Fig. 5c) and 1M-Mx^FLAG-NLS^ (Fig. 5d).

### EMcapsulin expression in mouse brain

To assess EMcapsulin contrast in mammalian neurons, we co-expressed 2M-Qt^FLAG^ with mScarlet-I in mouse hippocampus via viral transduction.

**Fig. 6:**
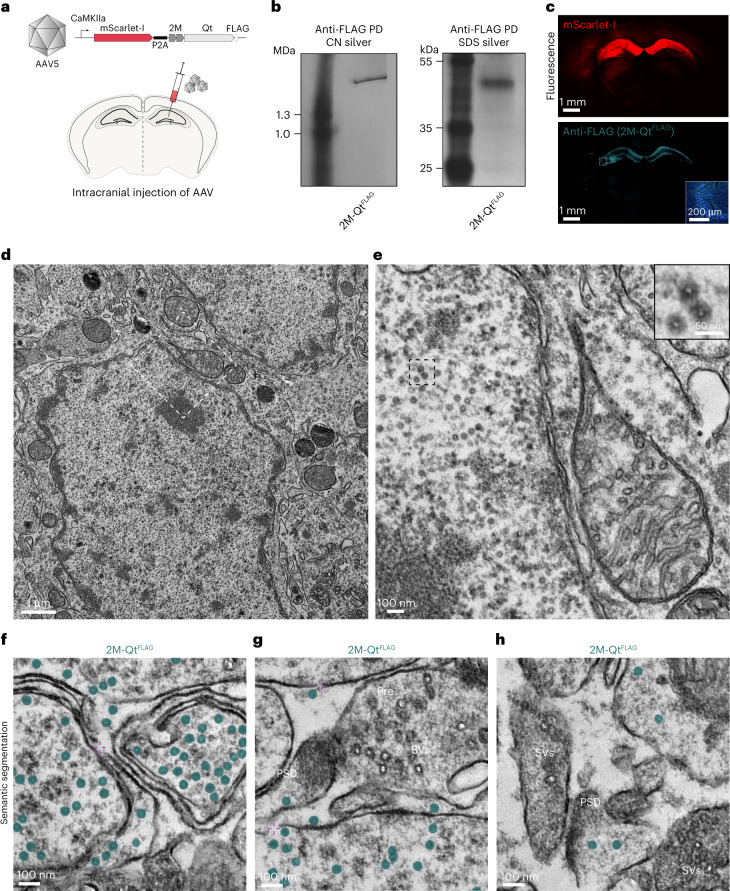
EMcapsulin expression in mouse brain. **a**, Schematic of the genetic construct for expressing 2M-Qt^FLAG^ together with mScarlet-I via AAV transduction and intracranial injection of AAVs into the hippocampus of a mouse. **b**, Native (CN) and corresponding SDS gels after silver staining confirming the assembly of 2M-Qt^FLAG^ after pull-down (PD) from the excised hippocampus. **c**, Confocal fluorescence imaging of a coronal section through the mouse brain 1 month after AAV transduction, showing direct mScarlet-I fluorescence in red and EMcapsulin expression in cyan (anti-FLAG, FITC). Scale bars, 1 mm and 200 µm (inset), respectively. The inset shows a zoomed-in region in the hippocampus. **d**,**e**, Overview TEM micrograph (**d**) and magnification of the region bounded via the white dashed lines in **d** (**e**). The inset shows a further zoom-in to the three EMcapsulins located inside the bounding box (black dashed lines). Scale bars for the respective magnifications are 1 μm, 100 nm and 50 nm. **f**–**h**, Instances of EMcapsulins in neuronal processes. The overlaid semantic segmentations are color-coded as defined in Fig. 1. ‘⊹’ denotes membrane discontinuities. Scale bars, 100 nm.

Native gel analysis verified EMcapsulin expression and assembly (Fig. 6b). Immunohistochemical fluorescence analysis confirmed EMcapsulin expression in CamKIIa-positive neurons in the hippocampus (Fig. 6c)^45^.

**Extended Data Fig. 7:**
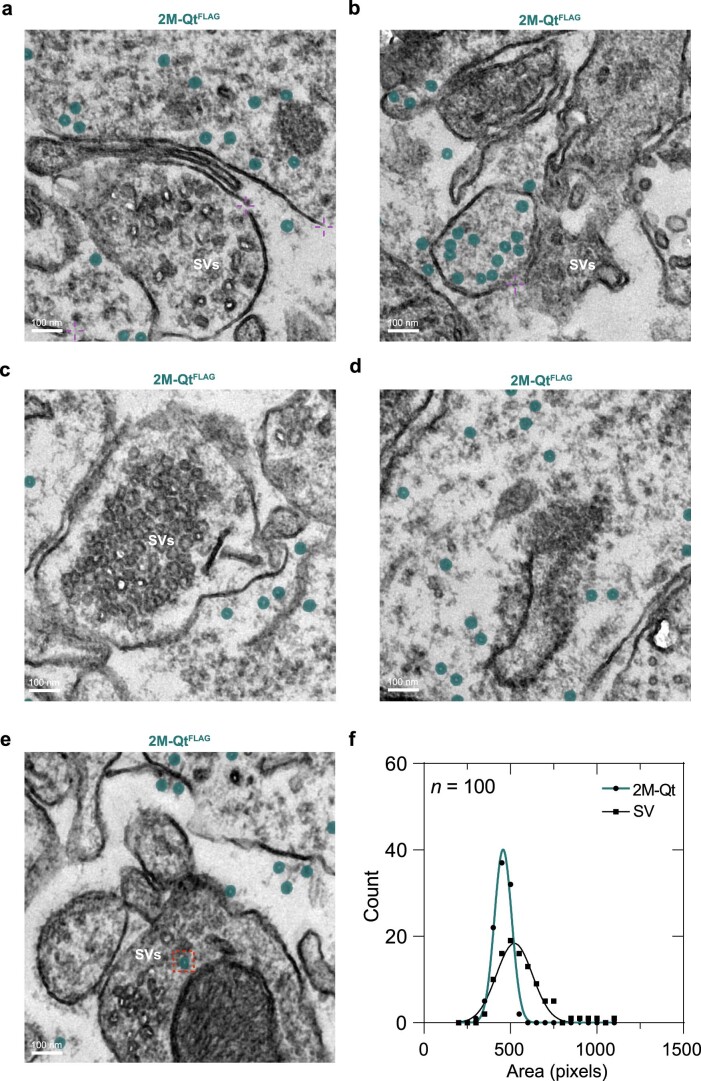
Additional TEM micrographs from EMcapsulins expressed in mouse hippocampus. Overlayed semantic segmentation maps (color coded as defined in Fig. 1, and related to Fig. 6) for 2M-Qt^FLAG^ in neuronal processes and in the vicinity of synaptic vesicles (SV). The scale bars represent 100 nm. Please note that the misclassification of an SV as an EMcapsulin, as shown in sub-panel e is very rare. EMcapsulins are rounder than synaptic vesicles resulting in clear annular contrast with concentric round borders on both the outer and inner diameter. Synaptic vesicles, on the other hand, have quite variable shapes in their cross sections consistent with their flexible lipid membranes, resulting in non-concentric inner and outer contrast boundaries. In 4 test images of processes in the hippocampus containing 251 manually annotated SVs, only 1 was misclassified as an EMcapsulin. **⊹** denotes membrane discontinuities. The scale bars represent 100 nm. **f**, Distribution of the areas of 2M-Qt^FLAG^ EMcapsulin particles and synaptic vesicles, *n*=100. 2M-Qt^FLAG^: 457.5 ± 50.05 pixels, SVs: 523.0 ± 104.1 pixels (mean±SD). Source data

EM analysis revealed EMcapsulin expression in neuronal somata (Fig. 6d, e) and processes (Fig. 6f–h), although some membrane discontinuities were observed in some regions, occasionally precluding a clear delineation of cellular boundaries. The narrow size distribution and sphericity of the rigid proteinaceous EMcapsulin shells produced concentric contrast edges at the inner and outer circumferences of the annular cross-sections. This appearance differentiated the EMcapsulins from synaptic vesicles, which exhibited more variable sizes and noncircular luminal and external borders consistent with their flexible lipid membranes (Extended Data Fig. 7).

Jointly, these imaging data show robust detection of barcoded EMcapsulins in different cell types and EM modalities.

## Discussion

We present a series of barcoded EM gene reporters (EMcapsulins) compatible with established fixation and staining protocols and correlative fluorescence microscopy for high-throughput volume EM pipelines that are increasingly in demand in cell biology and connectomics.

While high-resolution TEM naturally has higher discriminatory power for subnanometer shape differences, we deliberately chose spherical contrast elements with a diameter of tens of nanometers to allow differentiation by SEM-based volume EM methods.

By concatenating variable copies of metallothioneins to the inner surface of differently sized nanospheres, we obtained spherically symmetric EMcapsulin barcodes, which are ideal for sequential TEM and SEM as they can be robustly identified also on cross-sections without the need for 3D reconstructions.

We demonstrate that six different concentric barcodes can be automatically segmented and classified. In addition, the subcellular localization of the respective EMcapsulins (for example, nuclear versus cytosolic) can serve as a differentiator. Furthermore, multiple EMcapsulin barcodes can be modularly assembled into distinct patterns using a set of rigid cross-linkers of defined length.

Thus, the resulting combinatorial space for geometric multiplexing with EMcapsulins is already similar in size to that of commonly used fluorescent proteins for spectral multiplexing.

To make EMcapsulin classification convenient, we provide a napari GUI, which allows for interactively evaluating semantic segmentations from a specified model (Supplementary Fig. 3). Adding further annotated data from other laboratories and TEM instruments should improve the performance and robustness we obtained from our relatively small current dataset (250 TEM images).

Expanding the palette of spherical nanocompartments to larger sizes, for example, based on capsid structures with larger triangulation numbers, is an attractive future option.

Genetically defined information can also be encoded via controlling the subcellular localization of EMcapsulins, similar to filling certain subcellular compartments with dAPEX2-generated DAB-polymers^9^ but with nanometer-precise barcode information.

Although EMcapsulin contrast does not require additional staining steps, incubation with substrates or nanoparticles, or illumination used in current protocols, it could be compatible with DAB-polymerization-based labeling techniques using APEX2, which can be targeted to the lumen of encapsulins^17^ (Extended Data Fig. 5c). EMcapsulin expression showed no toxicity in cell culture or *Drosophila* and mouse brains.

We have furthermore shown how EMcapsulins can be rendered fluorescent and targeted to subcellular structures of interest to enable (live-cell) fluorescent microscopy followed by EM analysis for correlative workflows.

Fluorescent EMcapsulins will be helpful for engineering variants that are preferentially directed to pre- or postsynaptic compartments. This could be achieved by equipping them with peptides mimicking cargo adaptors of axonal transport machinery^46^ or via piggybacking on locally translated messenger RNA via a P2A motif or possibly via intein-based self-excision^47^.

As an alternative to the direct fusions of small fluorescent proteins to the EMcapsulin monomer, an extensive range of proteins can be encapsulated as guest molecules^17^ to add additional contrasts or functionalities.

With the growing interest in volume EM of cultured cells and organoids and the increasing number of (partial) EM connectomes, we anticipate a growing interest in multichannel EM reporters, which could respond to activity-dependent promoters or cellular events related to synaptic plasticity.

Similar to iron-oxide-biomineralizing encapsulins in cryo-electron tomography^17^, heavy metal organizing EMcapsulins could also serve as fiducial markers to improve tomographic reconstructions from tilt series^48–50^ or be used for drift correction^51^.

Given the natural trend in biomedical science towards volumetric tissue imaging at the best available resolution, the barcoded EMcapsulin will be a valuable toolset to augment anatomical EM data with multiplexed gene reporter information.

## Methods

### Molecular biology

All DNA constructs were custom-synthesized by Integrated DNA Technologies or assembled from multiple fragments via HiFi assembly, traditional ligation cloning (using EcoRI and NotI sites), or PCR-based mutagenesis methods, and cloned into pcDNA 3.1(+) Zeocin for mammalian expression. Supplementary Table 2 summarizes all genetic constructs used in this study.

### Mammalian cell culture

Low-passage-number HEK293T cells (ECACC: 12022001, obtained via Sigma-Aldrich) were cultured in Advanced Dulbecco’s modified Eagle medium with 10% FBS and penicillin–streptomycin at 100 µg ml^−1^ at 37 °C and 5% CO_2_. HEK293T cells were transfected using X-tremeGENE HP (Roche) transfection reagent according to the manufacturer’s protocol (3 µl reagent per microgram of DNA).

### Fly husbandry and strains

Flies were raised in the facilities at the Max Planck Institute for Biological Intelligence at 25 °C and 60% humidity on standard cornmeal agar medium at 12 h light/dark cycle. Only female brains were analyzed. The following driver lines were used: *elavC155-Gal4* (pan-neuronal expression, Bloomington Drosophila Stock Center (BDSC) 458), *R35A03-LexA* (expression in C3 neurons, BDSC 54706) and *R42F06-Gal4* (T4–T5 neuron expression, BDSC 41253). The UAS-EMcapsulin (1M-Qt^FLAG-NLS^ and 1M-Mx^FLAG–NLS^) strains were generated as follows: The DNA cassette encoding the EMcapsulins was custom-synthesized and subsequently cloned into XhoI/XbaI sites of pJFRC7-20XUAS-IVS-mCD8::GFP (Addgene, plasmid no. 26220), after removal of the mCD8::GFP cassette. The plasmids were injected into the attP2 landing site strain (BDSC, no. 8622) for PhiC31 integrase-mediated transgenesis (BestGene). The LexAop-EMcapsulin (3M-Qt^FLAG-NLS^) strain was generated as follows: The DNA cassette encoding the EMcapsulin was custom-synthesized and subsequently cloned pJFRC19-13XLexAop2-IVS-myr::GFP (Addgene, plasmid no. 26224) using XhoI/XbaI after removal of the myr::GFP cassette. For the expression of the 3M-Qt^FLAG-NLS^ and 1M-Mx^FLAG-NLS^ EMcapsulins in C3 and T4–T5 neurons, respectively, we used both the Gal4/UAS and LexA/LexAop binary expression systems. The final genotype allowed for the simultaneous expression of LexAop-3M-Qt^FLAG-NLS^ by the C3-neuronal driver *35A03-LexA* and the expression of UAS-1M-Mx^FLAG-NLS^ by the T4–T5-neuronal driver *R42F06-Gal4*. In a separate experiment, we pan-neuronally (*elavC155-Gal4* driver*)* co-expressed UAS-1M-Qt^FLAG-NES^ and UAS-1M-Mx^FLAG-NLS^.

### Experiments with mice

All in vivo experiments in mice were approved by the government of Upper Bavaria. Experiments were carried out in three male C57BL/6N, 3-month-old mice. Animals were housed in individually ventilated cages in specific-pathogen-free conditions and a 12 h light/dark cycle. Water and food were provided ad libitum.

#### Surgical preparations

Mice were administered 0.1 mg kg^−1^ buprenorphine (Temgesic, Indivior UK) intraperitoneally 30 min before the start of the surgery. Isoflurane was used for inhalation anesthesia: 5% for induction and 1.5–2% for maintenance. Anesthesia depth was checked by corneal and toe pinch reflexes, and the surgery started once these reflexes were absent. Body temperature was maintained around 36.5 °C with a heating mat, and corneal hydration was ensured using eye ointment (Bepanthen, Bayer).

#### Viral injections

Mice were positioned in a stereotaxic frame, and the skin was disinfected with Betadine (Braunoderm, Braun). Twenty microliters of lidocaine 2% (Braun) was injected subcutaneously for additional local skin and periosteum anesthesia. A 10-mm-long scalp incision was made, and the fascia was gently pushed to the side. The skull was cleaned and allowed a few minutes to dry. A 400-μm-diameter burr hole was drilled while avoiding overheating or damage to the meninges. A 33 gauge stainless steel injection cannula was inserted about 1,500 μm below the surface of the cortex, and 1 μl of AAV5-CaMKIIa-mScarlet-I-P2A-2M-Qt^FLAG^ (~1 × 10^12^ particles) solution was injected over a 10 min period using a syringe pump (PHD 22/2000, Harvard Apparatus). The cannula was held in position for 5 min after the injection to allow the viral solution to diffuse in the brain tissue and then slowly retracted. The incision was closed with tissue glue (Vetbond, 3 M), and lidocaine 2% was applied to the skin to prevent postoperative pain. For postoperative analgesia, 5 mg kg^−1^ meloxicam (Metacam 2 mg ml^−1^, Boehringer Ingelheim) was injected subcutaneously, and the animals were kept on a heated mat until they woke up. A total of 5 mg kg^−1^ meloxicam was administered subcutaneously once a day for the two subsequent days to provide postoperative analgesia.

#### Brain dissection

One month after the virus injection, mice were killed with an overdose of ketamine/xylazine and perfused with PBS. The brains were dissected and stored in 4% PFA solution for further processing for immunohistochemical analysis or directly homogenized in a mammalian protein extraction reagent (M-PER, Thermo Scientific, 78501) using a Dounce tissue homogenizer without fixation agent for pull-down experiments.

### Gel electrophoresis

Blue native (BN)- and CN-PAGE analyses were performed using the NativePAGE Novex Bis-Tris Gel System (Invitrogen) according to the protocol of the manufacturer.

For CN PAGE, the cathode buffer contained 0.05% of the anionic detergent sodium deoxycholate. Briefly, cell lysate volumes containing 100–500 ng of nanocompartments were loaded onto precast NativePAGE 3 to 12% gels and run at 150 V for 2 h at room temperature. If fluorescent protein assemblies were separated on CN PAGE, the apparatus was shielded from light to avoid bleaching. CN-PAGE gels were illuminated on a standard UV table and documented using a conventional cell phone camera to detect fluorescently labeled protein assemblies. To stain for the total protein content in the cell lysates, we performed a Coomassie staining on the BN/CN-PAGE gels. For on-gel APEX2-mediated DAB-polymer formation, gels were treated with DAB and hydrogen peroxide using the SIGMAFAST DAB Kit (Sigma-Aldrich, D0426). SDS–PAGE was performed using a Bio-Rad Mini-PROTEAN cell and precast 12% Bio-Rad TGX gels (40 min at 200 V). Protein bands from pull-down experiments were visualized using SilverQuest Silver Staining Kit (Invitrogen, LC6070). For pull-downs of FLAG-tagged EMcapsulins, anti-FLAG M2 magnetic beads (Sigma-Aldrich, M8823) were used according to the manufacturer’s protocol and eluted using 3× FLAG Peptide (Sigma-Aldrich, F4799). BC2-tagged EMcapsulins were pulled down with Spot-Trap Magnetic Agarose beads (ChromoTek, ‘etma’) according to the manufacturer’s protocol and eluted in the native state using an alkaline elution buffer.

### Immunolabeling and confocal microscopy (*Drosophila*)

For immunolabeling, brains were dissected in cold PBS and fixed in 4% paraformaldehyde (containing 0.1% Triton X-100) at room temperature for 22 min. Afterward, the brains were washed three times with PBT (PBS containing 0.3% Triton X-100) and blocked with 10% normal goat serum in PBT at room temperature for 1 h. Brains were then incubated with primary antibodies diluted in PBT containing 5% normal goat serum for 24–48 h at 4 °C.

After five wash steps with PBT, brains were incubated with secondary antibodies diluted in PBT containing 5% normal goat serum for 24–48 h at 4 °C. Brains were subsequently washed five times with PBT and once with PBS before being mounted in SlowFade Gold Antifade Mountant (Thermo Fisher Scientific). Imaging was performed using a Leica SP8 laser scanning confocal microscope equipped with 488, 561 and 633 nm lasers and a 63× objective. Image processing was performed with the ImageJ software package^52^. The following antibodies were used. Primary antibodies: rat anti-FLAG (1:200, Novus Biologicals, NBP-1-06712), rabbit anti-Sox102F (1:200 (ref. ^53^)), and mouse anti-Bruchpilot (1:20, Developmental Studies Hybridoma Bank (DSHB), AB2314866). Secondary antibodies (used at 1:400): Alexa Fluor 568-conjugated goat anti-mouse (Invitrogen, A11004), Alexa Fluor 568-conjugated goat anti-rabbit (Invitrogen, A-11011) and Alexa Fluor 647-conjugated goat anti-rat (Invitrogen, A21247). DAPI (1:1,000; Invitrogen) was applied for 5 min at the end of the immunolabeling protocol to stain nuclei, followed by extensive washing with PBT. The identity of adjacent C3 and T4–5 neurons in the immunohistochemistry (IHC) shown in Fig. 4f was established on the basis of the co-staining against Sox102f.

### Mouse brain IHC and microscopy

Brain tissue was cut into 70-µm-thick slices on a cryotome before incubation for 1 h at room temperature in a 1% BSA and 0.2% Triton X-100 in PBS solution to ensure permeabilization/blocking. Next, tissue slices were incubated at room temperature for 2 h with an anti-FLAG FITC (Sigma-Aldrich, F4049) monoclonal antibody conjugate diluted to a 2 μg ml^−1^. Slices were washed three times for 5 min in PBS and incubated with 10 mM DAPI. Again, tissue was washed three times for 5 min with PBS, and brain slices were mounted on microscopy slides with Aqua Poly Mount (Polysciences). Imaging was performed on a Leica SP5 (Leica Microsystems) to acquire the FITC signal corresponding to 2M-Qt^FLAG^ EMcapsulins and the signal from co-expressed mScarlet-I and DAPI.

### Fluorescence microscopy on HEK cells

Widefield fluorescence microscopy was performed on an EVOS fluorescence microscopy system (Invitrogen) equipped with filter cubes to image DAPI, GFP and RFP. The GFP filter cube was used to image eUnaG. A Leica SP5 (Leica Microsystems) equipped with 405, 488, 561 and 633 nm laser lines were used for confocal microscopy imaging, shown in Fig. 3. Colocalization analysis was performed with the Coloc2 plugin (release 3.0.5) for ImageJ (v1.53u). The region of interest was set around the prominent cells to exclude background. A PSF of 3.0 px was assumed as a conservative estimation for standard confocal microscopes. Background subtraction was performed in ImageJ on both channels with a 50 px rolling ball subtraction without smoothing. Costes *P* value was calculated with 100 randomizations.

### Live-cell microscopy on mammalian oocytes

Oocytes were isolated from the ovaries of 8- to 12-week-old FVB/N female mice. Fully grown oocytes of around 75 μm in diameter with a centered nucleus were arrested at prophase in homemade phenol red-free M2 supplemented with 250 μM dibutyryl cyclic AMP (Sigma-Aldrich) under paraffin oil (ACROS Organics) at 37 °C. For eUnaG imaging, the medium was supplemented with 3 µm bilirubin (Sigma-Aldrich). *eUnaG-1M-Qt*^*anti-mCherry*^, *mCherry-Myo5b*, *mCherry-Plk1* and *mCherry-RAB11A* mRNAs were synthesized and quantified as previously described^54^. Mouse oocytes were microinjected with 3.5 pl of mRNAs. *eUnaG-1M-Qt*^*anti-mCherry*^ mRNA was microinjected at a needle concentration of 112 or 224 ng µl^−1^, *mCherry-Myo5b* mRNA at 152.1 ng µl^−1^, *mCherry-PLK1* mRNA at 111.1 ng µl^−1^ and *mCherry-RAB11A* mRNA at 84.1 ng µl^−1^. Oocytes were allowed to express the mRNAs for 3 h before confocal or Airyscan imaging on LSM880 (Zeiss).

Automated segmentations of RAB11A-positive recycling endosomes and Myo5b-positive vesicles were performed using the Machine Learning Trainer function of Vision4D (Arivis). Segmented objects were tracked using the Tracking function. Specific parameters used were: Brownian Motion (Centroid) for motion type, 1 µm for maximum distance, Center of Geometry for centroid, no fusions or divisions, none for continue tracks, 2 for maximum time gap and none for weighting. The volumes and speeds of RAB11A-positive recycling endosomes and Myo5b-positive vesicles were then exported into Excel (Microsoft) and OriginPro (OriginLab).

### EM sample preparation

For EM sample preparation, the staining method was adapted from ref. ^55^ and used for both HEK cells and freshly dissected *Drosophila* brains. HEK293T cells were collected with Accutase (Sigma-Aldrich) 36 h post-transfection and pelleted by centrifugation. As an initial demonstration for multiplexed EMcapsulin detection across different cells, cell suspensions of HEK293T cells transiently expressing a single class were mixed and pelleted by centrifugation. Following the initial preparation, the material was fixed with 2.5% glutaraldehyde (Electron Microscopy Sciences) in 0.1 M sodium cacodylate buffer (pH 6.7–7.0) for 20 min (for *Drosophila* brains, the fixation duration was varying between 20 min and 24 h). After removal of the fixative, the material was postfixed using a 1:1 mixture of 4% OsO_4_ (Electron Microscopy Sciences) with 0.3 M sodium cacodylate buffer, containing 3% potassium hexacyanoferrate (II) (Sigma-Aldrich) for 20 min on ice. The postfixative solution was removed, and the material was washed twice with 0.1 M sodium cacodylate buffer for 2 min, followed by 2 × 5 min ddH_2_O washing steps. ddH_2_O water was removed, and the material was stained using 1% tannic acid (Sigma-Aldrich) for the duration of 10 min at room temperature. Then, tannic acid was decanted, and the material was washed five times for 5 min using ddH_2_O water. Subsequently, the material was treated with 1% UA (Electron Microscopy Sciences) solution (30 min, room temperature), with successive ddH_2_O 5 × 5 min washing. The material was then treated with 3% lead aspartate (Sigma-Aldrich) for 15 min at room temperature. Again, the material was washed (5 × 5 min in ddH_2_O water) before proceeding to epoxy embedding. The epoxy medium for the embedding process was prepared as follows: 61.5 g 2-dodecenylsuccinic-acid anhydride (Serva) was mixed with 81.5 g of methyl nadic anhydride (Serva) as well as with 130.5 g glycidether 100 (Serva). The resulting mixture was stirred, and 3,750 µl of 2,4,6-tris(dimethylaminomethyl)phenol (Serva) was added to the mixture, stirred and aliquoted for storage at −20 °C. The epoxy embedding process was conducted over 2 days. Immediately after the last washing step, the material was incubated on ice with 75% EtOH for 10 min, followed by incubation in 90% EtOH for another 10 min. Then, the sample was left in absolute ethanol for 1 h on ice with the solvent replacement after 30 min. The EtOH solution was replaced with pure propylene oxide and incubated for 5 min at room temperature. Next, the solution was discarded, and the material was incubated in a 1:1 mixture of epon and propylene oxide (Electron Microscopy Sciences) for 30 min under room temperature. Subsequently, the material was incubated at room temperature in 100% epon for 30 min and left in fresh epon for another 12 h. Epoxy was removed, and the material was briefly rinsed in fresh 100% epoxy and left in newly poured 100% epoxy for 72 h at 60 °C. The *Drosophila* brain before final epoxy curation was oriented such that optical lobes were parallel to the block’s slicing plane. The resulting blocks were subjected to trimming and slicing. For cellular material, the trimming of excess epoxy from the block’s surface was done using an EM TRIM milling system (Leica Microsystems). Using an UltraCut E microtome (Reichert/Leica) the prepared blocks were prepared with a histo-knife (DIATOME) and then sequentially cut with an ultra-knife (DIATOME) at a slice thickness of 70 nm, verified by the slices’ interference pattern. The slices were deposited either on the surface of a 200 mesh copper grid or the polished side of a silicon wafer.

### TEM

TEM images were acquired on a Libra120 TEM (Carl Zeiss GmbH), equipped with a CCD camera (TRÖNDLE Restlichtverstärkersysteme) using ImageSP software (SYSPROG). Before image acquisition, all grid-supported specimens were pre-irradiated at 120 kV beam voltage and 200 µrad illumination angle without apertures. The actual image acquisition took place with the activated BIO-AIS condenser aperture system and a 60 µm objective aperture. The same beam conditions used for pre-irradiation were also applied for imaging, except for an illumination angle of 100 µrad. The magnification for most of the TEM images was chosen such that a pixel size of 1.81 nm was achieved at an exposure time of 1,000 ms.

### SEM

SEM images were acquired using a Gemini 360 scanning electron microscope (Carl Zeiss GmbH), equipped with a sense-BSD detector (Carl Zeiss GmbH). Silicon wafers, supporting the samples were glued to the stage with silver glue (PLANO GmbH) and loaded on a sample holder. SEM image acquisition was performed with a 6.5 kV beam voltage, 2 nm nominal pixel size and 30 µm objective aperture, at a working distance of 3.7 mm. The stage was subjected to a bias voltage of 5 kV.

### FIB-SEM

FIB-SEM images were acquired on an SEM Crossbeam 550 (Carl Zeiss GmbH), equipped with InLens, BSE and SESI detectors (Carl Zeiss GmbH) running ATLAS software. The block with the specimen of interest was firmly attached to the stage using silver glue (PLANO GmbH). The sample was coated with an electron-transparent carbon layer (approximately 5 nm thickness) using an external carbon evaporator device. The carbon-coated sample was loaded into the FIB-SEM chamber, and a platinum guiding pad was deposited on the identified region of interest to aid the localization of the region of interest after sputter coating.

The sample was retrieved and loaded into an external sputter coater, where an electron-opaque iridium layer (~30 nm thickness) was deposited. Back in the FIB-SEM chamber, a 3D platinum pad with a thickness of 2 µm was placed onto the block in proximity to the region of interest. Subsequently, a tracking pattern was milled in the deposited pad to simplify image registration. Finally, the platinum pad and grooves were covered with the carbon layer (2 µm thickness) on top. The following settings were used for image acquisition: SEM beam voltage 1.3 kV, a working distance of 5 mm, 6 µs target dwell time and 4 nm nominal voxel size using an InLens detector. The FIB Ga beam was accelerated by 30 kV voltage at a current of 700 pA. An image volume of 3,696 nm (width) × 1,956 nm (height) × 404 nm (milling length) was acquired in ~70 min using ATLAS 3D software (Carl Zeiss GmbH). The acquired volume was pre-aligned by template matching to the surface landmarks and post-aligned with linear stack alignment using the SIFT algorithm implemented in ImageJ^56^. Renderings of the acquired FIB-SEM data were computed either with Imaris (Imaris 9.8 Oxford Instruments PLC) or with Dragonfly software (Dragonfly 2021.3, Object Research Systems).

### End-to-end multiclass semantic segmentation network

We employed a basic U-Net architecture^26^ inspired by Falk and colleagues^57^. For our training runs, we used a dropout of 0.1 and mish activation function^58^ and otherwise used the defaults of the MONAI implementation (https://docs.monai.io/en/stable/networks.html#basicunet). This implementation represents a standard U-Net architecture with an encoder and decoder connected by skip connections and has been proven successful in other biomedical segmentation tasks^59–62^. The network features one input and eight output channels. Besides an output channel for each of the six EMcapsulin classes, we implemented a background channel and a channel for the EMcapsulin patterns consisting of cross-linked 1M-Qt and 1M-Tm (Fig. 2). A percentile-based normalization is applied for training and inference^63^.

#### Training

We used 250 TEM micrographs (pixel size of 1.81 nm) taken on a Libra120 TEM (Carl Zeiss GmbH). We trained the model for 3,000 epochs with Ranger21 optimizer^64^, an initial learning rate of 1 × 10^−2^, and a batch size of 2. A batch consisted of 20 random crops sized 512 × 512 pixels. During training, we employed basic augmentation strategies, namely Gaussian noise, flips and random affine transformations. An equally weighted sum of soft Dice and binary cross entropy inspired by Isensee et al.^63^ served as a loss function for our training runs.

#### Inference

To derive segmentations, we combined test time augmentations, namely flips and Gaussian noise, with a sliding window inference. For the sliding window inference, we used a batch size of 32 and an overlap of 0.5. We derive multiclass segmentation maps by computing argmax on the six class channels. Further, we provide the possibility to preserve network outputs for all eight channels enabling downstream analysis.

#### Postprocessing

To refine the segmentation maps, we conducted conservative postprocessing in a multistep procedure and provided means to fine-tune each of the steps on an individual basis. Therefore, we first binarize the six-channel segmentation maps. Then we compute a connected component analysis on the binarized segmentation maps using cc3d (ref. ^65^). We then remove particles with fewer than 42 pixels because EMcapsulins are at least 20 nm in diameter (~96 pixels area). We chose this threshold value as a compromise to remove ‘noise’ but maintain partially successful segmentations (for example, half-rings on the borders of EMcapsulins). Next, we conducted conditional majority voting within each binary connected component. We thus assigned the class represented by the majority of pixels. This step was applied only if the structures were below a maximum of 500 pixels to refrain from modifying the class of touching objects and a circularity larger than 0.2. We also refrained from the majority voting in case of ties (several majority classes with the same number of pixels). Ultimately, we filled up holes in the multiclass segmentation maps for pixels that were completely surrounded by foreground pixels belonging to the same class.

#### Datasets and annotation

An intra-image split into training (70% of the raw image) and validation set (30% of the raw image) was performed by randomly choosing respective ‘stripes' in each TEM micrograph. Pixel-accuracy annotations were performed in ImageJ, yielding a total number of 35,282 annotated particles in the training dataset. Independently acquired TEM micrographs were annotated for the independent test set 1 (57 images, 10 images annotated for the subset ‘two EMcapsulins classes in adjacent HEK cells’) and test set 2 (single EMcapsulin class, 26 images, 14 images annotated).

#### Evaluation

Besides qualitative analysis, we relied on quantitative metrics for the comparison of our convolutional neural network models. Therefore, we report pixel-wise DSC, sensitivity and precision. Furthermore, we computed instance-level metrics based on an intersection-over-union criterion of 0.5. We further report the three panoptic quality metrics^27^ (Supplementary Table 1).

We summed up the respective confusion matrices globally for the computation of both pixel and instance metrics across all microscopy slices.

We conducted this procedure to treat all EMcapsulins equally, irrespective of their occurrence, in a dense or sparse microscopy image. We approximated instances with a connected component analysis using cc3d (ref.^65^) since our annotations were not optimized for instance semantic segmentation.

This heuristic is not perfect, as closely located EMcapsulins touching each other can be merged into one instance. Therefore, factually correct network predictions might be classified as false positives resulting in overly pessimistic instance-level metric computations. On the basis of the above criteria, we select the checkpoint from epoch 2070, producing the lowest loss and, coincidentally, also the best volumetric DSC for training. We report results on the test set 1 in Figs. 1d, 2c, 3g, 4g and 6f–h, Supplementary Figs. 5 and 7c,d and Extended Data Fig. 7, showing multiclass segmentation maps as an overlay.

#### Hardware

Computations were run on a rack server equipped with an AMD EPYC 7313 16-Core Processor in combination with NVIDIA RTX 8000 and A5000 GPUs using CUDA version 11.4 in conjunction with Pytorch 1.13.0 and MONAI version 1.0.

### Sequential segmentation-classification pipeline

The model was implemented in PyTorch using the elektronn3 neural network toolkit (https://github.com/ELEKTRONN/elektronn3) and trained and tested on NVIDIA A40 GPUs, hosted at the Max Planck Computing facility MPCDF in Garching, Germany. The same 250 TEM micrographs with intra-image splits were also used for training and validation of the segmentation-classification pipeline. The U-Net model for segmentation was enhanced by including an additional batch normalization layer^66^ after each convolution layer and trained for 160,000 steps using the AdamW (ref. ^67^) optimizer and a batch size of 8. To mitigate the impact of the strong foreground-to-background class imbalance of the training data, the training objective was chosen to be the sum of a weighted Dice loss function^68^ and a weighted cross-entropy loss function (foreground pixels were weighted five times more than background pixels). The following augmentations were applied during training of the U-net segmentation model: cropping of 384 × 384 pixel patches from random regions of the source images, random flipping, shifting, scaling and rotation, additive Gaussian noise, random gamma correction, and random brightness and contrast changes.

The EfficientNetV2-M model for patch-based EMcapsulin classification was trained with random flipping, scaling and rotation augmentations. It was trained for 120,000 steps using the AdamW optimizer with a batch size of 128. The dataset of patch images was rebalanced by undersampling overrepresented classes. In order to prevent the model from fitting onto potentially informative background information in the vicinity of the EMcapsulins, the background was locally masked from the patches by setting all pixels to 0 values that did not belong to the foreground segmentation mask produced by the U-Net. To filter out falsely merged neighboring EMcapsulin segmentation instances and irregularly shaped segmentation masks, the circularity, defined as $4 \times \pi \times {\mathrm{area}}/{\mathrm{perimeter}}^2$, of each connected-component instance was calculated. Segmentation instances below an area of 60 or above 2,304 pixels, and instances touching the image borders were not considered for classification. Additionally, objects with a circularity below 0.8 were not classified. To construct the majority-vote-based confusion matrices, we sampled n particles from the test set and assigned them the most frequently occurring class, as determined by the EfficientNetV2-M.

A napari GUI was developed in Python to enable interactive segmentation, classification and visualization of TEM images, as shown in Supplementary Fig. 6.

### Reporting summary

Further information on research design is available in the Nature Portfolio Reporting Summary linked to this article.

## Online content

Any methods, additional references, Nature Portfolio reporting summaries, source data, extended data, supplementary information, acknowledgements, peer review information; details of author contributions and competing interests; and statements of data and code availability are available at 10.1038/s41587-023-01713-y.

## Supplementary information

Supplementary Information Supplementary Figs. 1–8 and Table 1.

Reporting Summary

Supplementary Video 1 Time-lapse Airyscan movie of a mouse oocyte microinjected with mCherry-RAB11A mRNA but no eUnaG-1M-Qt^anti-mCherry^. Green channel with the same settings as in the other videos; magenta: mCherry-RAB11A.

Supplementary Video 2 Time-lapse Airyscan movie of a mouse oocyte microinjected with 112 ng µl^−1^ eUnaG-1M-Qt^anti-mCherry^ and mCherry-RAB11A mRNAs. Green: eUnaG-1M-Qt^anti-mCherry^; magenta: mCherry-RAB11A.

Supplementary Video 3 Time-lapse Airyscan movie of a mouse oocyte microinjected with 224 ng µl^−1^ eUnaG-1M-Qt^anti-mCherry^ and mCherry-RAB11A mRNAs. Green: eUnaG-1M-Qt^anti-mCherry^; magenta: mCherry-RAB11A.

Supplementary Video 4 Time-lapse Airyscan movie of a mouse oocyte microinjected with mCherry-Myo5b mRNA but no eUnaG-1M-Qt^anti-mCherry^. Green channel with the same settings as in the other videos; magenta: mCherry-Myo5b.

Supplementary Video 5 Time-lapse Airyscan movie of a mouse oocyte microinjected with 224 ng µl^−1^ eUnaG-1M-Qt^anti-mCherry^ and mCherry-RAB11A mRNAs. Green: eUnaG-1M-Qt^anti-mCherry^; magenta: mCherry-Myo5b.

Supplementary Video 6 Rendering of FIB-SEM data of EMcapsulins expressed in *Drosophila* neurons.

Supplementary Table 1 Performance metrics for the end-to-end multiclass semantic segmentation model.

Supplementary Table 2 Amino acid sequences of all constructs used in this study.

## Source data

Source Data Fig. 2 Unprocessed photo of the CN-PAGE gel shown in Fig. 2b. Raw distances values in nanometers of the bar graph shown in Fig. 2d.

Source Data Extended Data Fig. 1 Unprocessed photo of the CN-PAGE gel shown in Extended Data Fig. 1a. Unprocessed photo of the SDS–PAGE gel shown in Extended Data Fig. 1b. Intensity values shown in the radial plot profiles in Extended Data Fig. 1d for the five replicates (image level) for which the *t*-test was calculated at the distance 9.85 nm.

Source Data Extended Data Fig. 3 Uncropped photo of the CN-PAGE gel shown in Extended Data Fig. 3b.

Source Data Extended Data Fig. 4 Unprocessed photo of the Coomassie-stained CN-PAGE gel shown in Extended Data Fig. 4a. Unprocessed photo of the UV-illuminated CN-PAGE gel shown in Extended Data Fig. 4b (same gel as shown in 4a). Uncropped version of the silver-stained SDS-PAGE shown in Extended Fig. 4c from which densitometric analyses were conducted. Densitometric integrals from which the bar graphs in Extended Data Fig. 4d were created.

Source Data Extended Data Fig. 5 Unprocessed photo of the Coomassie-stained CN-PAGE gel shown in Extended Data Fig. 5c. Unprocessed photo of the DAB/H_2_O_2_-treated CN-PAGE gel shown in Extended Data Fig. 5c.

Source Data Extended Data Fig. 6 Uncropped version of the exemplary silver-stained SDS–PAGE shown in Extended Fig. 6a from which densitometric analyses determining the rt ratios were performed. Numeric values for rt efficiencies in Extended Fig. 6b.

Source Data Extended Data Fig. 7 Raw surface area pixel values of 2M-Qt^FLAG^ particles and synaptic vesicles found in TEM micrographs from mouse hippocampal regions expressing mScarlet-I_P2A_2M-Qt^FLAG^ from which the size distributions in Extended Fig. 6f were generated.

## Data Availability

All sequences of the genetic constructs are available (Supplementary Table 2). TEM data are provided together with the code. FIB-SEM volumes will be available from the corresponding author upon request. Source data are provided with this paper.

## Code availability

Source code for the end-to-end multiclass segmentation network is available at https://github.com/ggwlab/EMcapsulins and for the sequential segmentation-classification pipeline at https://github.com/StructuralNeurobiologyLab/emcaps.
