# Supplementary material for: Genetically encoded barcodes for correlative volume electron microscopy
Source: Nat Biotechnol. 2023 Apr 17;41(12):1734–45. doi: 10.1038/s41587-023-01713-y (PMC10713455; doi:10.1038/s41587-023-01713-y)
Supplement: Supplementary file 1 — Supplementary Figs. 1–8 and Table 1. [file 41587_2023_1713_MOESM1_ESM.pdf]

nature biotechnology

Article

https://doi.org/10.1038/s41587-023-01713-y

Genetically encoded barcodes for correlative volume electron microscopy

In the format provided by the authors and unedited

Table of Contents

Supplementary Figure 1 | High-res TEM micrographs of EMcapsulins expressed in HEK293T. - page 2

Supplementary Table 1 | Performance metrics for the end-to-end multi-class semantic segmentation model. - page 2

Supplementary Figure 2 | Performance metrics for sequential segmentation and classification pipeline. - page 3

Supplementary Figure 3 | Napari GUI for interactive segmentation and classification of EMcapsulins. - page 4

Supplementary Figure 4 | Live microscopy experiments with targeted fluorescent EMcapsulins. - pages 5,6

Supplementary Figure 5 | EMcapsulins targeted to subcellular compartments in mammalian cells and *Drosophila* cells. - page 7

Supplementary Figure 6 | Higher-magnification TEM micrographs of EMcapsulins expressed in *Drosophila* neurons. - page 7

Supplementary Figure 7 | Detection of EMcapsulins in the processes of *Drosophila* optic lobe neurons. - page 8

Supplementary Figure 8 | Normalized radial profiles of EMcapsulins from comparative TEM and SEM images - page 9

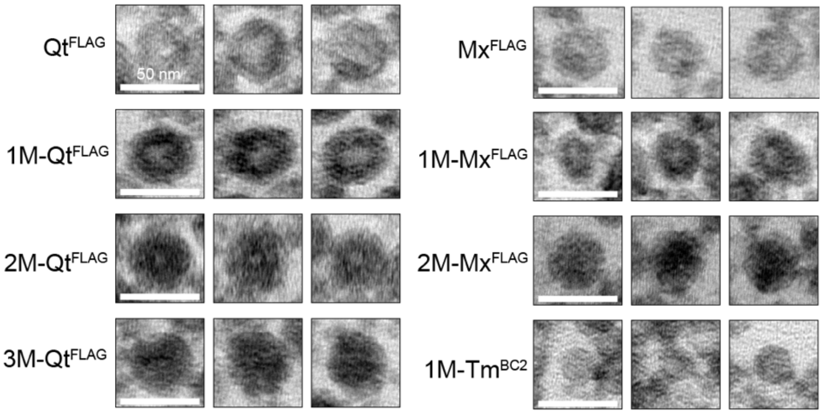

Supplementary Figure 1 | High-res TEM micrographs of EMcapsulins expressed in HEK293T.
All six classes of EMcapsulins and wild-type controls (Qt^{FLAG}, Mx^{FLAG}) were acquired at a target image pixel size of 0.23 nm and an illumination angle of 0.800 mrad. The scale bars are 50 nm.

Dataset	Class	DSC	S	P	RQ	SQ	PQ	IS	IP
**Validation Set (single EMcapsulin class)**	1M-Qt	0.84 (0.84)	0.87 (0.87)	0.80 (0.80)	**0.78** (0.75)	0.86 (0.86)	**0.67** (0.64)	0.78 (0.78)	**0.78** (0.72)
	2M-Qt	0.69 (0.69)	0.76 (0.76)	0.63 (0.63)	**0.73** (0.70)	0.82 (0.82)	**0.60** (0.57)	0.78 (0.78)	**0.69** (0.63)
	3M-Qt	0.70 (0.70)	0.63 (0.63)	0.79 (0.79)	**0.66** (0.64)	0.83 (0.83)	**0.55** (0.53)	0.56 (0.56)	**0.79** (0.73)
	1M-Mx	0.65 (0.65)	0.66 (0.66)	0.64 (0.64)	**0.64** (0.59)	0.82 (0.82)	**0.52** (0.48)	0.64 (0.64)	**0.63** (0.55)
	2M-Mx	0.66 (0.66)	0.66 (0.66)	**0.67** (0.66)	**0.65** (0.58)	0.85 (0.85)	**0.55** (0.49)	0.65 (0.65)	**0.65** (0.53)
	1M-Tm	0.41 (0.41)	0.34 (0.34)	0.52 (0.52)	**0.40** (0.37)	0.79 (0.79)	**0.31** (0.29)	0.33 (0.33)	**0.50** (0.42)
**Test Set 1 (two EMcapsulins classes in adjacent HEK cells)**	1M-Qt	0.90 (0.90)	0.92 (0.92)	0.89 (0.89)	**0.87** (0.83)	0.94 (0.94)	**0.82** (0.79)	**0.89** (0.88)	**0.85** (0.79)
	2M-Qt	0.80 (0.80)	0.74 (0.74)	0.86 (0.86)	**0.79** (0.76)	0.94 (0.94)	**0.74** (0.71)	0.76 (0.76)	**0.83** (0.76)
	3M-Qt	0.61 (0.61)	0.49 (0.49)	0.80 (0.80)	**0.61** (0.58)	0.87 (0.87)	**0.54** (0.50)	0.52 (0.52)	**0.75** (0.65)
	1M-Mx	0.64 (0.64)	**0.51** (0.50)	0.87 (0.87)	**0.64** (0.60)	0.90 (0.90)	**0.58** (0.54)	0.53 (0.53)	**0.81** (0.70)
	2M-Mx	0.72 (0.72)	0.87 (0.87)	0.61 (0.61)	**0.70** (0.62)	0.88 (0.88)	**0.62** (0.54)	0.86 (0.86)	**0.59** (0.48)
	1M-Tm	0.61 (0.61)	0.63 (0.63)	**0.59** (0.58)	**0.59** (0.54)	**0.59** (0.48)	**0.45** (0.42)	**0.67** (0.66)	**0.53** (0.46)
**Test Set 2 (single EMcapsulin class)**	1M-Qt	0.82 (0.82)	0.87 (0.87)	0.77 (0.77)	**0.72** (0.69)	0.83 (0.83)	**0.60** (0.57)	0.69 (0.69)	**0.77** (0.69)
	2M-Qt	0.86 (0.86)	0.80 (0.80)	0.94 (0.94)	0.88 (0.88)	0.88 (0.88)	0.78 (0.78)	0.86 (0.86)	0.90 (0.90)
	3M-Qt	0.42 (0.42)	0.27 (0.27)	0.92 (0.92)	**0.39** (0.36)	0.82 (0.82)	0.32 (0.29)	0.26 (0.26)	**0.78** (0.63)
	1M-Mx	0.65 (0.65)	0.60 (0.60)	0.71 (0.71)	**0.63** (0.58)	0.78 (0.78)	0.45 (0.45)	0.54 (0.54)	**0.74** (0.62)
	2M-Mx	0.81 (0.81)	0.86 (0.86)	0.76 (0.76)	**0.77** (0.74)	0.89 (0.89)	**0.69** (0.65)	0.85 (0.85)	**0.71** (0.65)
	1M-Tm	0.27 (0.27)	0.24 (0.24)	0.31 (0.31)	**0.28** (0.26)	0.80 (0.80)	**0.23** (0.21)	0.27 (0.27)	**0.30** (0.26)

Supplementary Table 1 | Performance metrics for the end-to-end multi-class semantic segmentation model.
We report pixel-level metrics, namely Dice similarity coefficients (DSC), sensitivity (S), and precision (P). Additionally, instance-level metrics are displayed: Recognition quality (RQ), Segmentation quality (SQ), Panoptic quality (PQ), instance-sensitivity (IS), and instance-precision (IP). The confusion matrix for the instance-level metrics is computed based on a panoptic IoU ≥ 0.5 criterion. Values before (in parentheses) and after post-processing. Changes due to post-processing are displayed in bold. Note that this table is also supplied as .xls file.

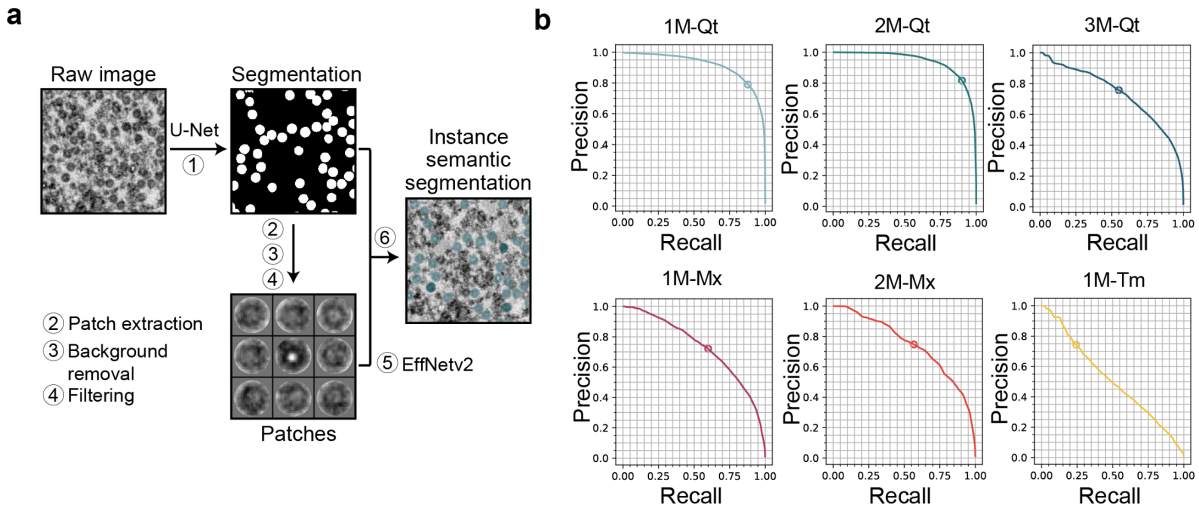

c

EMcapsulins class	Precision	Recall	DSC	IoU
1M-Qt	0.79	0.89	0.84	0.72
2M-Qt	0.6	0.88	0.71	0.56
3M-Qt	0.83	0.67	0.74	0.59
1M-Mx	0.71	0.59	0.64	0.48
2M-Mx	0.75	0.56	0.64	0.47
1M-Tm	0.74	0.24	0.36	0.22

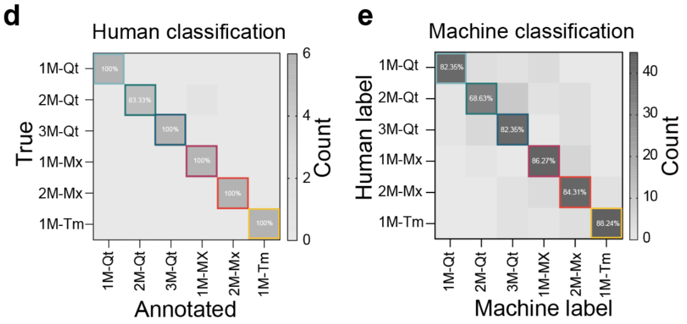

Supplementary Figure 2 | Performance metrics for sequential segmentation and classification pipeline.
a, Flowchart describing the two-step semantic instance segmentation pipeline consisting of segmentation of EMcapsulins and background subtraction, followed by classification of the EMcapsulin instances. **b**, Pixel-level Precision-recall (PR) curves for all 6 EMcapsulin classes, as shown in Figure 1b. **c**, Tabulated segmentation metrics for all 6 EMcapsulin classes. Overall average model precision is 82.2%, recall is 74.6%, DSC is 0.78, and IoU is 0.64. **d**, Confusion matrix of a human classification experiment where 3 blinded human evaluators classified 12 patches of TEM micrographs from HEK293T cells (400 x 400 pixels, 2 examples per EMcapsulins class) to one of the six different EMcapsulin classes (average accuracy: 97.22 %). **e**, Confusion matrix for the classification results of the EfficientNetV2 on the segmented EMcapsulins.

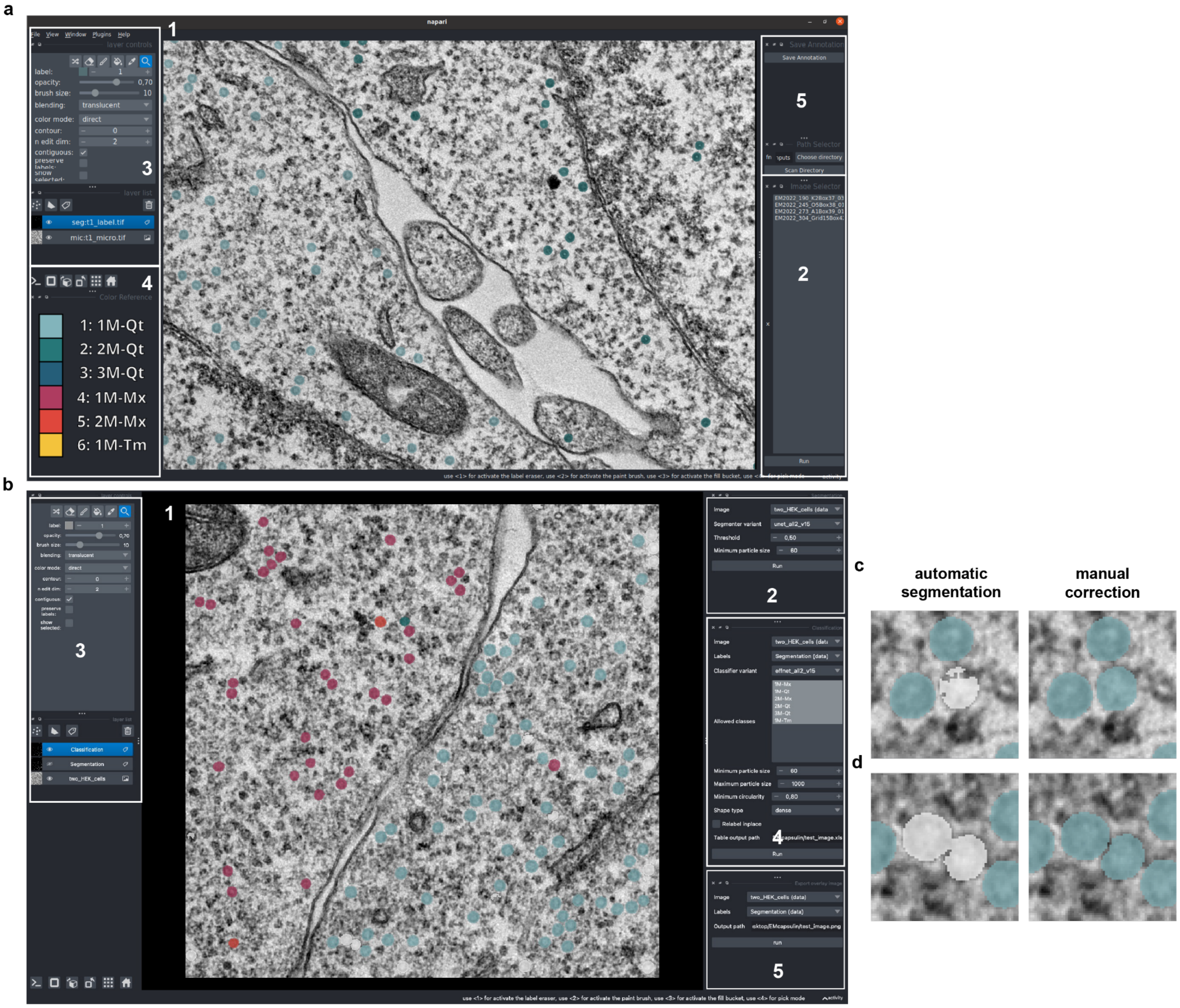

Supplementary Figure 3 | Napari GUIs for interactive segmentation and classification of EMcapsulins.
a, Screenshot of the napari GUI for the end-to-end semantic segmentation network, which allows for interactive label curation and interfacing with the U-net model (https://github.com/ggwlab/EMcapsulins). (1) Napari image viewer, (2) import widget for loading all images as segmentation results in a selected folder, (3) napari tools for manual annotation, (4) color-code for the EMcapsulin classes, (5) export widget for the curated masks. **b,** Screenshot of the napari GUI for the sequential segmentation-classification pipeline (https://github.com/StructuralNeurobiologyLab/emcaps): (1) napari image viewer, (2) segmentation widget (3) napari tools for manual annotation (4) classification widget (5), export widget for exporting a semantic segmentation overlay. **c**,**d** Examples of possible automated segmentation results flagged for not meeting the imposed criteria (linear pixel size range and circularity). Manual curation of incomplete or artificially fused segmentation instances (middle panel) allows for an improved class assignment (right).

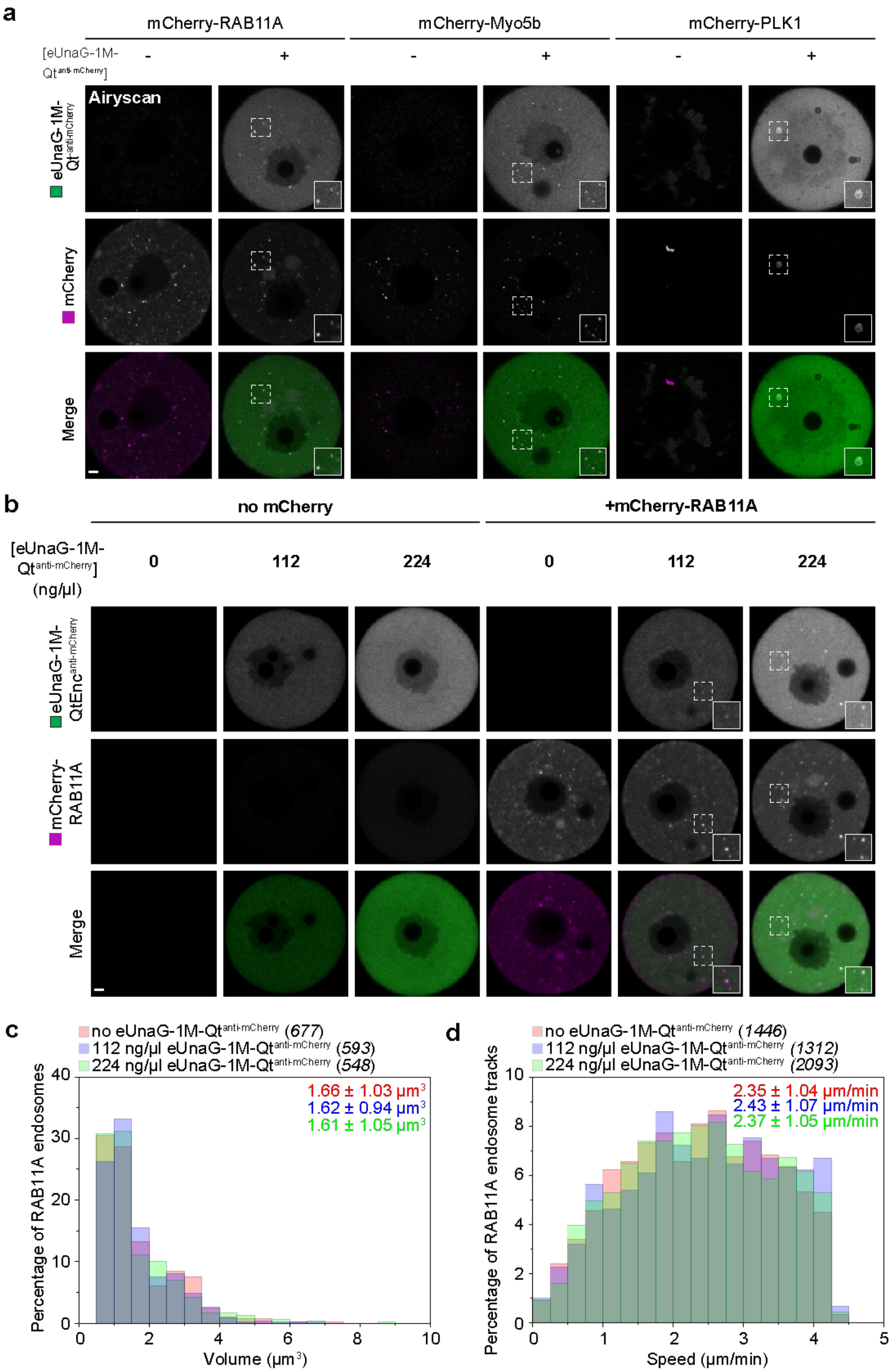

Supplementary Figure 4 | Live microscopy experiments with targeted fluorescent EMcapsulins.
a, Individual frames from live-cell microscopy (Airyscan LSM880) of mammalian oocytes (**Supplementary Videos 1-5**) co-expressing mCherry-RAB11A, Myo5b-mCherry, or mCherry-PLK1 without or with eUnaG-1M-Qt$^{\text{anti-mCherry}}$. Insets are magnifications of regions outlined by dashed boxes. The scale bar represents 5 µm. **b**, Confocal images of live mouse oocytes microinjected with different concentrations of eUnaG-1M-Qt$^{\text{anti-mCherry}}$ mRNA in the absence and presence of mCherry-RAB11A. **c,d,** Quantification of volume (c) and speed (d) of RAB11A-positive recycling endosomes in mCherry-RAB11A-expressing mouse oocytes microinjected with different concentrations of eUnaG-1M-Qt$^{\text{anti-mCherry}}$ mRNA. The number of particles or tracks analyzed is specified in italics.

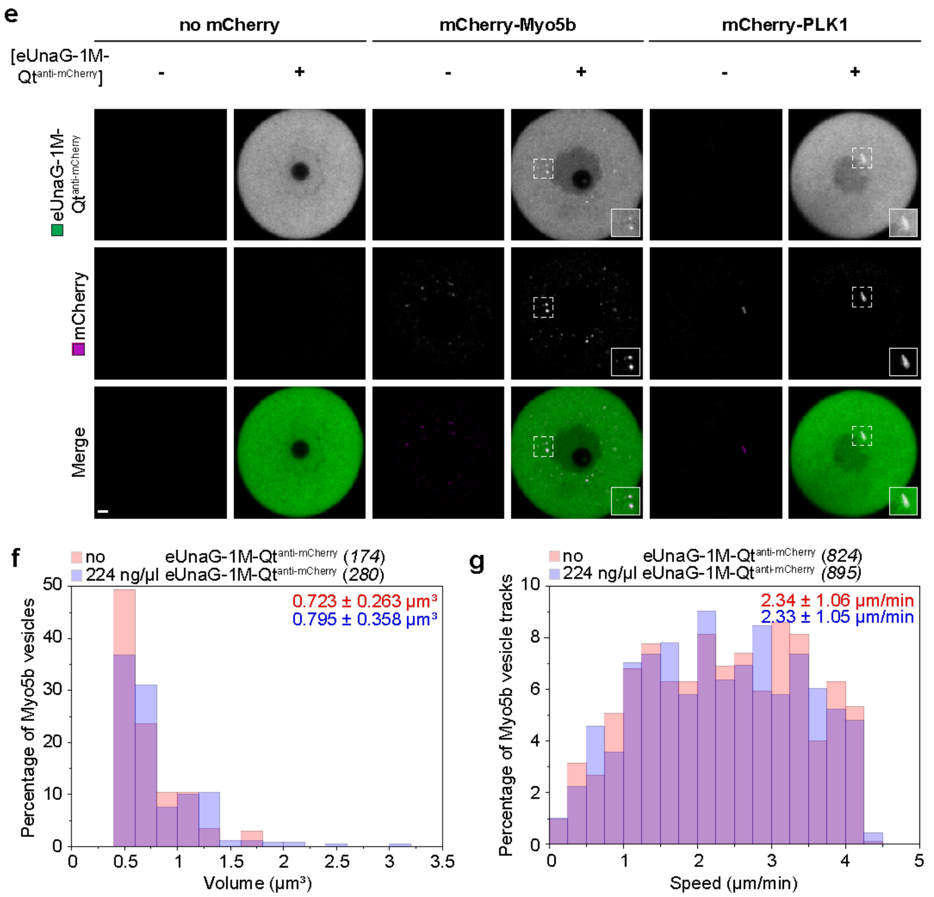

Supplementary Figure 4 continued |

e, Confocal images of live mouse oocytes expressing no mCherry target, mCherry-Myo5b, or mCherry-PLK1 in the absence and presence of eUnaG-1M-Qt$^{anti\text{-}mCherry}$ (224 ng/µl mRNA). Insets are magnifications of the regions outlined by the dashed boxes. Scale bars are 5 µm. **f,g,** Quantification of volume (f) or speed (g) of mCherry-Myo5b-positive vesicles.

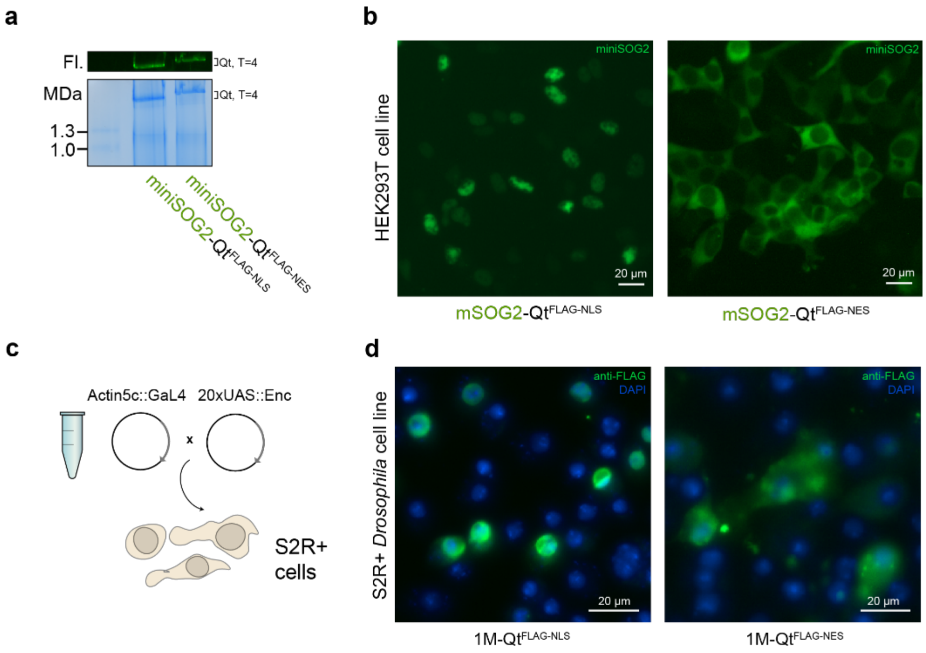

Supplementary Figure 5 | EMcapsulins targeted to subcellular compartments in mammalian cells and *Drosophila* cells.
a, UV-fluorescence and Coomassie-stained BN-PAGE of lysates from HEK293T cells expressing QtEnc N-terminally fused to the small fluorescent protein miniSOG2, and C-terminally fused to a FLAG-epitope followed by an NLS (cMyc) or an NES (HIV). The bands apparent above 1.3 MDa correspond to the native assembled nanocompartments. The bands also show fluorescence, indicating that the N-terminally appended miniSOG2 was functional and did not comprise the assembly. **b**, Corresponding live-cell fluorescence microscopy showing miniSOG2-Qt$^{FLAG\text{-}NLS}$ predominantly localized in the nucleus and miniSOG2-Qt$^{FLAG\text{-}NES}$ predominantly localized in the cytosol. **c**, The functionality of NLS constructs was confirmed in the S2R+ *Drosophila* cell line by co-expressing either UAS-1M-Qt$^{FLAG\text{-}NLS}$ or UAS-1M-Qt^{FLAG} with Actin5c-Gal4. **d**, Widefield fluorescence microscopy showed that EMcapsulins with the NLS downstream of the FLAG-epitope (anti-FLAG, green signal) readily co-localized with the nucleus (DAPI, blue signal), whereas the variant without NLS was distributed throughout the cytosol.

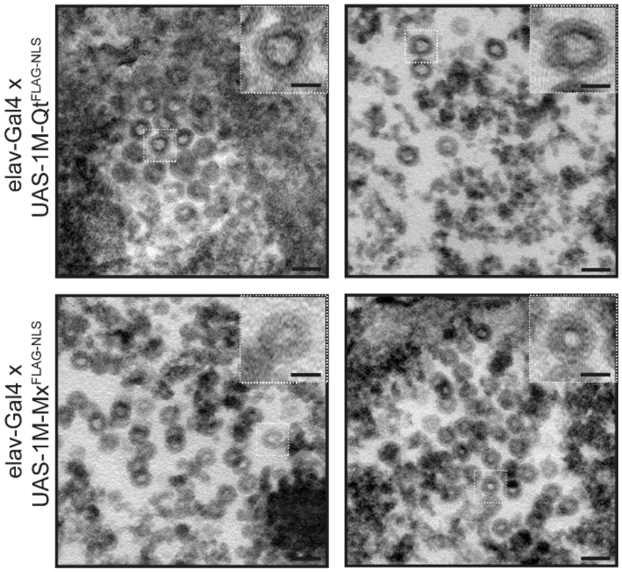

Supplementary Figure 6 | Higher-magnification TEM micrographs of EMcapsulins expressed in *Drosophila* neurons.
Neurons in the optic lobe expressing 1M-Qt$^{FLAG\text{-}NLS}$ (top) or 1M-Mx$^{FLAG\text{-}NLS}$ (down). Scale bars are 50 nm (20 nm for inset).

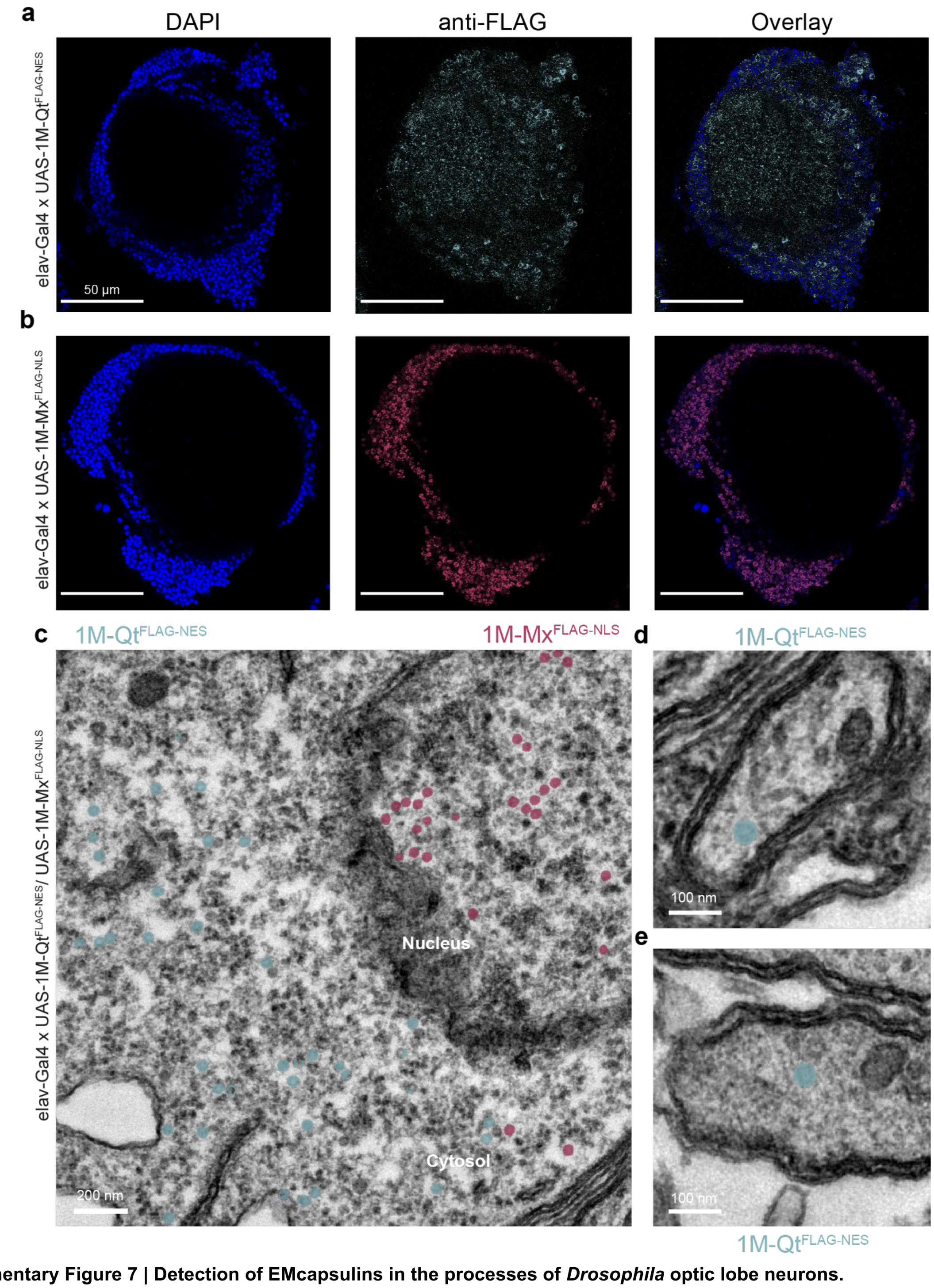

Supplementary Figure 7 | Detection of EMcapsulins in the processes of *Drosophila* optic lobe neurons.
a, b, IHC of the optic lobe (OL) of *Drosophila* brains with pan-neuronal expression of either 1M-Qt$^{FLAG-NES}$ (a) or 1M-Mx$^{FLAG-NLS}$ (b). Anti-FLAG (Alexa 568, cyan for 1M-Qt$^{FLAG-NES}$ and red for 1M-Mx$^{FLAG-NLS}$) and DAPI (blue) to show the distribution of the neuronal nuclei. Note that there is a substantial anti-FLAG signal in the neuropil only if the nuclear export signal (NES) but not the nuclear localization signal (NLS) is expressed. The scale bars represent 50 µm. **c,** TEM micrograph of the optic lobe of *Drosophila* pan-neuronally co-expressing 1M-Qt$^{FLAG-NES}$ and 1M-Mx$^{FLAG-NLS}$ with the multi-class semantic segmentation results overlaid according to the color scheme in main Figure 1. 1M-Mx$^{FLAG-NLS}$ EMcapsulins are predominantly found in the nucleus. 1M-Qt$^{FLAG-NES}$ EMcapsulins can be detected in the cytosol as well as in projections (d,e). Scale bar in sub-panel c represents 200 nm. The scale bars in d and e represent 100 nm.

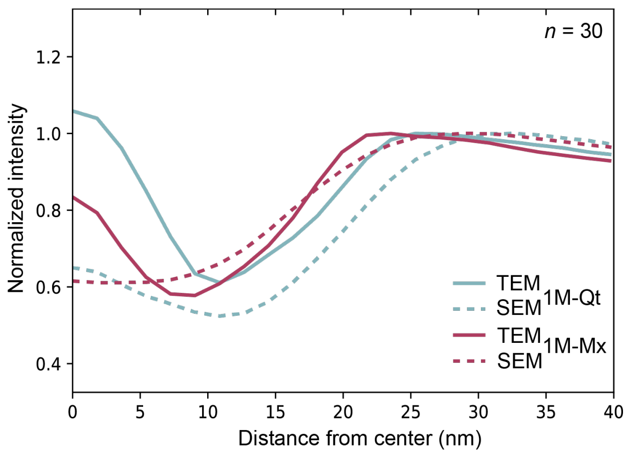

Supplementary Figure 8 | Normalized radial profiles of EMcapsulins from comparative TEM and SEM images. These radial profiles were computed from the TEM and SEM images shown in Figure 5 a,b.
