# Supplementary material for: Genetically encoded barcodes for correlative volume electron microscopy
Source: Nat Biotechnol. 2023 Apr 17;41(12):1734–45. doi: 10.1038/s41587-023-01713-y (PMC10713455; doi:10.1038/s41587-023-01713-y)
Supplement: Supplementary file 2 — Reporting Summary [file 41587_2023_1713_MOESM2_ESM.pdf]

Corresponding author(s): Gil Westmeyer

Last updated by author(s): Feb 4, 2023

Reporting Summary

Nature Portfolio wishes to improve the reproducibility of the work that we publish. This form provides structure for consistency and transparency in reporting. For further information on Nature Portfolio policies, see our Editorial Policies and the Editorial Policy Checklist.

Statistics

For all statistical analyses, confirm that the following items are present in the figure legend, table legend, main text, or Methods section.

n/a	Confirmed	
☐	☒	The exact sample size (*n*) for each experimental group/condition, given as a discrete number and unit of measurement
☐	☒	A statement on whether measurements were taken from distinct samples or whether the same sample was measured repeatedly
☐	☒	The statistical test(s) used AND whether they are one- or two-sided<br>*Only common tests should be described solely by name; describe more complex techniques in the Methods section.*
☐	☒	A description of all covariates tested
☐	☒	A description of any assumptions or corrections, such as tests of normality and adjustment for multiple comparisons
☐	☒	A full description of the statistical parameters including central tendency (e.g. means) or other basic estimates (e.g. regression coefficient) AND variation (e.g. standard deviation) or associated estimates of uncertainty (e.g. confidence intervals)
☐	☒	For null hypothesis testing, the test statistic (e.g. F, t, r) with confidence intervals, effect sizes, degrees of freedom and P value noted<br>*Give P values as exact values whenever suitable.*
☒	☐	For Bayesian analysis, information on the choice of priors and Markov chain Monte Carlo settings
☒	☐	For hierarchical and complex designs, identification of the appropriate level for tests and full reporting of outcomes
☐	☒	Estimates of effect sizes (e.g. Cohen's *d*, Pearson's *r*), indicating how they were calculated

Our web collection on statistics for biologists contains articles on many of the points above.

Software and code

Policy information about availability of computer code

Data collection	TEM image aquisition: ImageSP software (SYSPROG, Belarus), (FIB)-SEM image aquisition: ATLAS software (Carl Zeiss GmbH, Germany)
Data analysis	Graphpad Prism 9 (Graphpad Software, Inc), Microsoft Excel, Origin Pro (OriginLab), Vision4D (Arivis), Excel (Microsoft), OriginPro (OriginLab), Customized Python code, ImageJ v1.53k, PyTorch using the elektronn3 neural network toolkit (https://github.com/ELEKTRONN/elektronn3), MONAI implementation of U-net (https://docs.monai.io/en/stable/networks.html#basicunet)

For manuscripts utilizing custom algorithms or software that are central to the research but not yet described in published literature, software must be made available to editors and reviewers. We strongly encourage code deposition in a community repository (e.g. GitHub). See the Nature Portfolio guidelines for submitting code & software for further information.

Data

Policy information about availability of data

All manuscripts must include a data availability statement. This statement should provide the following information, where applicable:

- Accession codes, unique identifiers, or web links for publicly available datasets
- A description of any restrictions on data availability
- For clinical datasets or third party data, please ensure that the statement adheres to our policy

All sequences of the genetic constructs will be made available. TEM data and FIB-SEM volumes will be available from the corresponding author upon request.

Human research participants

Policy information about studies involving human research participants and Sex and Gender in Research.

Reporting on sex and gender	n/a
Population characteristics	n/a
Recruitment	n/a
Ethics oversight	n/a

Note that full information on the approval of the study protocol must also be provided in the manuscript.

Field-specific reporting

Please select the one below that is the best fit for your research. If you are not sure, read the appropriate sections before making your selection.

☒ Life sciences ☐ Behavioural & social sciences ☐ Ecological, evolutionary & environmental sciences

For a reference copy of the document with all sections, see nature.com/documents/nr-reporting-summary-flat.pdf

Life sciences study design

All studies must disclose on these points even when the disclosure is negative.

Sample size	We found average intensity projections of EMcapsulins to be robust for >100 particles. Please see detailed information on data split and augmentation for the ML procedures in the method section.
Data exclusions	No data points were excluded.
Replication	Data shown in Figures 1-4 were successfully replicated at least once with independent biological samples. Some samples were imaged by different EM modalities.
Randomization	Cell culture wells were allocated to the different treatment groups specified by the particular experiment without determining any specific property of the cells in a given well for selecting a specific condition. The sample patches for the human classification were randomized.
Blinding	Samples prepared for EM were renamed by a running index and processed in parallel by a several researchers, such that a bias for a specific sample was made improbable. The human classifiers were blinded.

Reporting for specific materials, systems and methods

We require information from authors about some types of materials, experimental systems and methods used in many studies. Here, indicate whether each material, system or method listed is relevant to your study. If you are not sure if a list item applies to your research, read the appropriate section before selecting a response.

Materials & experimental systems

n/a	Involved in the study	
☐	☒	Antibodies
☐	☒	Eukaryotic cell lines
☒	☐	Palaeontology and archaeology
☐	☒	Animals and other organisms
☒	☐	Clinical data
☒	☐	Dual use research of concern

Methods

n/a	Involved in the study	
☒	☐	ChIP-seq
☒	☐	Flow cytometry
☒	☐	MRI-based neuroimaging

Antibodies

Antibodies used	mouse anti-FLAG M2 (F1804, Sigma-Aldrich), mouse anti-FLAG M2 FITC (F4049, Sigma Aldrich), rat anti-FLAG (1:200, Novus Biologicals, NBP-1-06712), rabbit anti-Sox102F (1:200) Schilling, T., Ali, A. H., Leonhardt, A., Borst, A. & Pujol-Martí, J. Transcriptional control of morphological properties of direction-selective T4/T5 neurons in Drosophila. Development 146, (2019), mouse anti-Bruchpilot (1:20, Developmental Studies Hybridoma Bank (DSHB), AB2314866). Secondary ABs: Alexa Fluor 568-conjugated goat anti-

	mouse (Invitrogen, A11004), Alexa Fluor 568-conjugated goat anti-rabbit (Invitrogen, A-11011), Alexa Fluor 647-conjugated goat anti-rat (Invitrogen, A21247)
Validation	All antibodies are commonly used and were purchased from respected manufacturers that present validation data on the respective product website.

Eukaryotic cell lines

Policy information about cell lines and Sex and Gender in Research

Cell line source(s)	HEK293T (human) (ECACC 12022001), S2R+ (Drosophila) (FlyBase ID: FBtc0000150)
Authentication	No further authentification method was performed.
Mycoplasma contamination	Mycoplasma contamination was monitored using the MycoAlert ® PLUS Mycoplasma Detection Kit (Lonza).
Commonly misidentified lines (See ICLAC register)	n/a

Animals and other research organisms

Policy information about studies involving animals; ARRIVE guidelines recommended for reporting animal research, and Sex and Gender in Research

Laboratory animals	Drosophila melanogaster (attP2 landing site strain (Bloomington Drosophila Stock Center, no. 8622), elavC155-Gal4 (pan-neuronal expression, Bloomington Drosophila Stock Center (BDSC) 458), R35A03-LexA (expression in C3 neurons, BDSC 54706), R42F06-Gal4 (T4-T5 neurons expression, BDSC 41253); C57BL/6N wild-type mice.
Wild animals	n/a
Reporting on sex	n/a
Field-collected samples	n/a
Ethics oversight	government of Upper Bavaria, Germany

Note that full information on the approval of the study protocol must also be provided in the manuscript.
